# Characterization of the African Swine Fever Virus Decapping Enzyme during Infection

**DOI:** 10.1128/JVI.00990-17

**Published:** 2017-11-30

**Authors:** Ana Quintas, Daniel Pérez-Núñez, Elena G. Sánchez, Maria L. Nogal, Matthias W. Hentze, Alfredo Castelló, Yolanda Revilla

**Affiliations:** a Centro de Biología Molecular Severo Ochoa, CSIC-UAM, Universidad Autónoma de Madrid, Madrid, Spain; b European Molecular Biology Laboratory (EMBL), Heidelberg, Germany; c Department of Biochemistry, University of Oxford, Oxford, United Kingdom; University of Southern California

**Keywords:** African swine fever virus, decapping, ribosomes, translation, virus-cell interaction

## Abstract

African swine fever virus (ASFV) infection is characterized by a progressive decrease in cellular protein synthesis with a concomitant increase in viral protein synthesis, though the mechanism by which the virus achieves this is still unknown. Decrease of cellular mRNA is observed during ASFV infection, suggesting that inhibition of cellular proteins is due to an active mRNA degradation process. ASFV carries a gene (Ba71V D250R/Malawi g5R) that encodes a decapping protein (ASFV-DP) that has a Nudix hydrolase motif and decapping activity *in vitro*. Here, we show that ASFV-DP was expressed from early times and accumulated throughout the infection with a subcellular localization typical of the endoplasmic reticulum, colocalizing with the cap structure and interacting with the ribosomal protein L23a. ASFV-DP was capable of interaction with poly(A) RNA in cultured cells, primarily mediated by the N-terminal region of the protein. ASFV-DP also interacted with viral and cellular RNAs in the context of infection, and its overexpression in infected cells resulted in decreased levels of both types of transcripts. This study points to ASFV-DP as a viral decapping enzyme involved in both the degradation of cellular mRNA and the regulation of viral transcripts.

**IMPORTANCE** Virulent ASFV strains cause a highly infectious and lethal disease in domestic pigs for which there is no vaccine. Since 2007, an outbreak in the Caucasus region has spread to Russia, jeopardizing the European pig population and making it essential to deepen knowledge about the virus. Here, we demonstrate that ASFV-DP is a novel RNA-binding protein implicated in the regulation of mRNA metabolism during infection, making it a good target for vaccine development.

## INTRODUCTION

Turnover of mRNA is not only important for regulation of cellular gene expression, it also plays an essential role in antiviral defense, since degradation of viral RNA is employed as part of the protective immune response (1–4). Thus, viruses have developed mechanisms to alter cellular RNA metabolism in order to block or subvert stress responses and facilitate viral replication (5, 6). Viruses can alter the distribution and quantity of cellular mRNAs by modifying cellular RNA metabolism functions, as is the case with herpes simplex virus 1 (HSV-1), adenovirus (AdV), vesicular stomatitis virus (VSV), poliovirus, and influenza virus (7–12). In other cases, viruses directly degrade mRNA via viral ribonucleases that induce cellular host shutoff, such as in alpha- and gammaherpesviruses, severe acute respiratory syndrome coronavirus (SARS-CoV), and influenza virus (13–17). Two families of viruses have been described that use direct decapping as part of their strategy to inhibit cellular translation and promote viral protein synthesis (18): the Saccharomyces cerevisiae double-stranded L-A virus, which uses a specific mechanism of decapping related to cap snatching (4), and vaccinia virus (VACV), which possesses two viral decapping enzymes (D9 and D10) (19, 20). These viral enzymes, which have approximately 25% sequence similarity, possess a Nudix motif, a highly conserved 23-amino-acid sequence (GX_5_EX_5_[UA]XREX_2_EEXGU, where X can be any amino acid and U is a hydrophobic residue). *In vitro* assays have demonstrated that both recombinant D9 and D10 proteins exhibit decapping activity, which is abrogated in both proteins by single-site mutations of the conserved glutamate residues in the Nudix motif, indicating that it is essential for catalysis (19, 20).

African swine fever virus (ASFV) is a large, cytoplasmic, double-stranded DNA virus that infects different species of swine, including domestic pigs and wild boar (21, 22). Morphogenesis of the virus takes place in specialized areas of the cytoplasm called viral factories, which are located near the nucleus and the microtubule organization center (MTOC). These structures essentially exclude cellular proteins but are surrounded by endoplasmic reticulum (ER) membranes and enclosed in vimentin cages (23).

ASFV exhibits notable transcriptional independence from its host, with 20% of its genome dedicated to genes related to transcription and mRNA modification (24). Consequently, ASFV infection is characterized by a fine-tuned temporal regulation of viral gene expression, which allows transcripts to be classified as immediate early, early, intermediate, or late depending on their distinctive accumulation kinetics and expression in the presence of inhibitors of viral replication, such as cytosine arabinoside (AraC) (25). Interestingly, the virus is able to temporally control the expression of its genes while simultaneously causing strong cellular shutoff (25–27). However, ASFV protein synthesis relies on the cellular translation machinery, with eukaryotic initiation factor 4E (eIF4E) and eIF4G playing a critical role (26).

ASFV carries a gene (D250R in strain Ba71V and g5R in strain Malawi) that encodes a decapping protein (ASFV-DP) that has a Nudix hydrolase motif and decapping activity *in vitro* (28). The Nudix motif is essential for catalysis, as single-site mutation of the conserved residues E147, E150, and E151 causes a loss of decapping activity. The Malawi strain isoform, g5Rp, is able to interact directly with RNA, and though its activity is strongly inhibited by uncapped RNAs, it is not affected by cap analogs or methylated nucleotides, suggesting that ASFV-DP preferentially recognizes the RNA moiety on the substrate (28). Nevertheless, the *in vivo* role of the protein is still unknown.

The Nudix motif of ASFV-DP displays sequence similarity to other decapping enzymes, such as Dcp2 (decapping enzyme 2), the yeast decapping enzyme (29), suggesting its direct involvement in mRNA regulation. Therefore, it is a good candidate to be the viral factor involved in degradation of cellular mRNAs and the temporal regulation of viral mRNAs. In this study, the role of ASFV-DP was investigated in cultured cells and during infection. At late stages of infection, ASFV-DP presents subcellular localization typical of the viral factories, where it colocalizes with the mRNA cap structure. Moreover, we show that it is able to interact with components of the ribosomes, such as the RPL23a protein, and with poly(A) RNA in cells stably expressing the protein. We have determined that this interaction is mediated primarily by the ASFV-DP N-terminal region and that conserved amino acids of the Nudix motif are not required for the interaction. This viral protein also interacts with viral and cellular RNAs in the context of infection, and its overexpression in infected cells results in decreased levels of both species of transcripts.

## RESULTS

### ASFV infection promotes a decrease in cellular mRNA levels.

**FIG 1:**
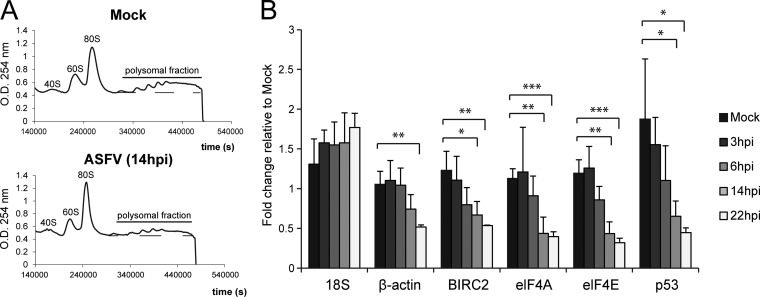
Effects of ASFV infection on cellular and viral translation. (A) Polysome profile analysis. Vero cells were mock or ASFV infected (MOI = 5 PFU/cell), and samples were recovered at 14 hpi in the presence of cycloheximide (100 μg/ml). Polysomes were fractionated in sucrose gradients and examined in a polygraph; densitometry values were read at 260 nm and are represented as a function of time. O.D., optical density. (B) Quantification of cellular mRNA levels during ASFV infection. Samples from mock- or ASFV-infected (MOI = 5 PFU/cell) Vero cells were recovered at different times postinfection. After RNA extraction, cellular mRNA levels of representative genes were measured by RT-qPCR. RNA values are means and SD (*n* = 3). *, *P* < 0.03; **, *P* < 0.01; ***, *P* < 0.001.

ASFV infection induces a strong cellular shutoff (26, 27), but the molecular mechanism by which this important regulatory step is achieved remains elusive. In order to determine if a defect in translation is involved, we analyzed the polysome profile of uninfected (mock-infected) and ASFV-infected Vero cells. Polysomes are mRNAs interacting with two or more ribosomes, and *de novo* protein synthesis is reflected in the number and amount of ribosomes found within the polysomal mRNA fraction (30). Mock- and ASFV-infected Vero cells (multiplicity of infection [MOI] = 5 PFU/cell) were lysed at 14 h postinfection (hpi) in the presence of cycloheximide (CHX), an inhibitor of protein synthesis that blocks elongation, causing ribosome stalling on mRNA (31), followed by resolution of polysomes in a sucrose gradient (30). By 14 hpi, viral protein synthesis is mainly achieved, whereas cells are still alive, allowing the analysis of the effect of viral infection on cell translation machinery events. ASFV infection promoted a reduction in the amount of polysomes (measured as the area under the line of the polysome fraction), but not in the polysome number (measured as the number of peaks found on the polysome fraction) (Fig. 1A), which suggests that ASFV infection triggers a decrease in the amount of mRNAs being translated. To further clarify this result, we next quantified the levels of some representative, unrelated cellular mRNAs by reverse transcription-quantitative PCR (RT-qPCR) during ASFV infection. A significant decrease in all analyzed cellular mRNAs was observed from 14 hpi (Fig. 1B), with similar timing to cellular shutoff. These results suggest that the ASFV-induced cellular shutoff may be at least partially due to an active degradation of cellular mRNA.

### ASFV encodes a protein harboring a canonical Nudix motif.

**FIG 2:**
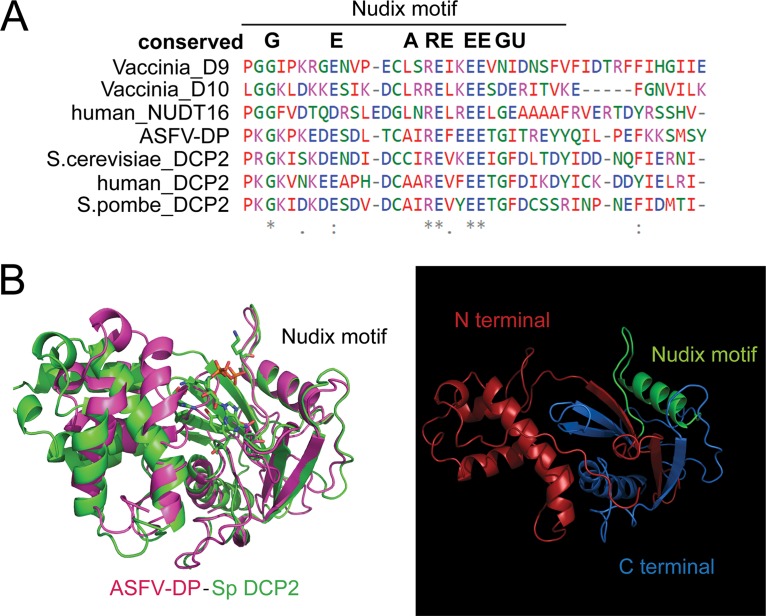
ASFV-DP contains a Nudix motif. (A) Partial sequence alignment of the ASFV-DP Nudix motif sequence with other conserved viral and eukaryotic Nudix amino acid sequences. Alignments were performed using Clustal-Ω software (EMBL). The colors represent the different amino acid classes: small and hydrophobic (red); acidic (blue); basic (magenta); hydroxyl, sulfhydryl, amine, or glycine (G) residues (green); and others (gray). The asterisks indicate positions that have a single, fully conserved residue; the colons indicate conservation of strongly similar properties; the periods indicate conservation between groups of weakly similar properties. (B) Structural prediction of the ASFV-DP protein based on Dcp2 of S. pombe. (Left) The ASFV-DP structure is shown in pink, and the SpDcp2 structure is shown in green. (Right) The ASFV-DP structure is represented alone. The N-terminal region, C-terminal region, and Nudix motif are shown in red, blue, and green, respectively.

Comparison of the ASFV-DP Nudix motif to other well-known decapping enzymes, such as the human decapping Dcp2 (hDcp2) and Nudt16 enzymes, Schizosaccharomyces pombe Dcp2 (SpDcp2) and S. cerevisiae Dcp2 (ScDcp2) of yeast, and the viral proteins D9 and D10 from VACV, revealed 16 to 21% similarity (Table 1 and Fig. 2A). Indeed, ASFV-DP not only possesses the amino acids that are highly conserved among Nudix motifs, it also shares predicted structural homology with the decapping enzymes compared. The ASFV-DP structure was modeled based on the SpDcp2 crystal structure (Fig. 2B), and maximal structural similarity was found in the helix structure of the catalytic Nudix motif, while the N- and C-terminal regions had lower similarities. The terminal regions of the Nudix motif are commonly implicated in regulation of decapping activity and are less conserved (18), in agreement with what is observed in ASFV-DP.

**TABLE 1:** Identity matrix of the ASFV-DP Nudix motif surrounding sequence compared to cellular and viral decapping enzymes

Protein	% identity with:						
	VACV D10	VACV D9	Human Nutd16	ASFV-DP	S. cerevisiae Dcp2	Human Dcp2	S. pombe Dcp2
VACV D10	100	23.79	15.44	16.49	18.81	12.87	18.81
VACV D9	23.79	100	17.12	19.19	18.82	14.62	19.77
Human Nutd16	15.44	17.12	100	17.61	16.77	16.35	18.87
ASFV-DP	16.49	19.19	17.61	100	19.63	16.44	21.46
S. cerevisiae Dcp2	18.81	18.82	16.77	19.63	100	26.96	29.86
Human Dcp2	12.87	14.62	16.35	16.44	26.96	100	37.5
S. pombe Dcp2	18.81	19.77	18.87	21.46	29.86	37.5	100

### ASFV-DP localizes around the viral factories at late times postinfection.

**FIG 3:**
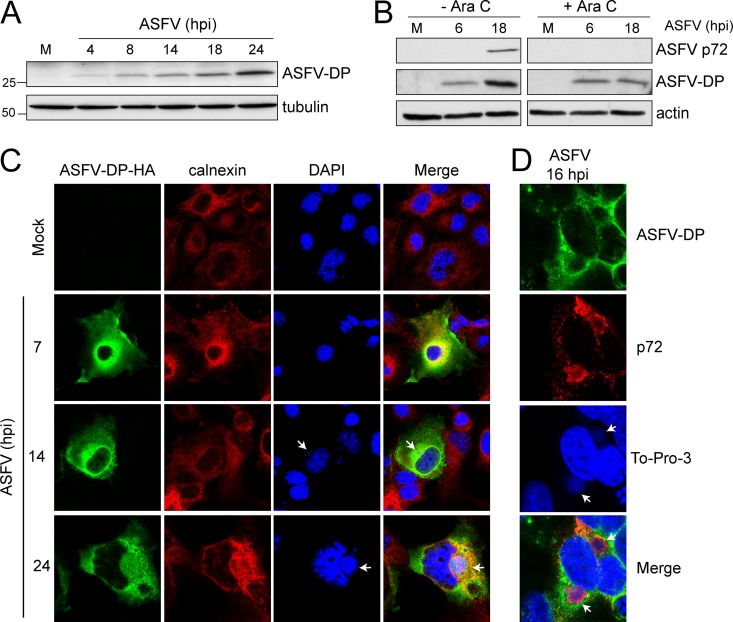
ASFV-DP expression and localization during ASFV infection. (A) ASFV-DP is expressed early during infection and accumulates until late times postinfection. At the indicated times, mock (M)- and ASFV-infected (MOI = 5 PFU/cell) Vero cells were lysed, and ASFV-DP levels were determined by Western blotting using specific anti-ASFV-DP serum; tubulin was used as a loading control. (B) Vero cells were mock or ASFV infected in the presence or absence of an inhibitor of viral replication, AraC (40 μg/ml). Samples were recovered at the indicated times to analyze ASFV-DP and p72 (capsid) protein levels by Western blotting. The late viral protein p72 was used to confirm the effect of AraC, and actin was used as a loading control. (C) COS-7 cells were transfected with pASFV-DP-HA, infected 12 h later with ASFV (MOI = 5 PFU/cell), and fixed at the indicated times postinfection. Calnexin and ASFV-DP were localized using specific anti-HA (green) and anti-calnexin (red) antibodies. The progress of the infection was tracked by visualizing viral factories and cellular DNA with DAPI (blue). Arrows indicate viral factories. (D) ASFV-DP localizes in the surroundings of the viral factories in ASFV-infected COS-7 cells at late times postinfection (16 hpi). ASFV-DP and p72 were localized using anti-ASFV-DP and anti-p72 specific sera (green and red, respectively).

In order to investigate the function of ASFV-DP during infection, mock- or ASFV-infected Vero cells were harvested at the indicated times postinfection and ASFV-DP levels were analyzed by Western blotting. A specific band of about 30 kDa was revealed as early as 4 hpi and accumulated until 24 hpi (Fig. 3A). In order to determine the temporal regulation of ASFV-DP expression, protein levels were assayed in mock- and ASFV-infected cells in the presence or absence of AraC (40 μg/ml) by Western blotting. ASFV-DP was detected in the presence of the drug, indicating that its synthesis does not require viral DNA replication, identifying ASFV-DP as an early viral protein (Fig. 3B). Furthermore, as shown in Fig. 3B, ASFV-DP was also detected at later times of the infection, a fact that can be related to the functional role of the protein. As a control, the late protein p72 was not detected in the presence of AraC (Fig. 3B).

To determine the subcellular localization of ASFV-DP, it was first cloned with a hemagglutinin (HA) tag under the control of the specific viral promoter in a pcDNA plasmid. COS-7 cells were transfected with pASFV-DP-HA and subsequently infected with ASFV in order to activate the viral promoter, allowing expression and localization in the context of natural ASFV infection. The progression of viral infection was assessed by the presence of viral factories, which, together with cellular DNA, were stained with DAPI (4′,6-diamidino-2-phenylindole) or To-Pro 3 (Thermo Fisher; T-3205) (blue) (Fig. 3C). We detected that ASFV-DP (green) presented a distribution pattern during the viral cycle similar to that of calnexin (red), a marker of the ER, indicating that ASFV-DP localizes to the organelle (Fig. 3C). Due to the fact that several mRNAs are translated on the surface of the ER (32), ASFV-DP localization on the organelle is compatible with its expected decapping activity, as the viral protein would have easier access to capped mRNAs. Interestingly, ASFV-DP was spread within the cytoplasm but accumulated at the viral factories (mainly at the peripheries of the structures) at late times postinfection, consistent with a possible role in the regulation of viral mRNA stability (Fig. 3C and D). It has been previously reported that viral membrane precursors are derived from the ER cisternae, which are recruited to the viral factory and later modified (33), which could explain the observed presence of both calnexin and ASFV-DP at the peripheries of viral replication sites at late times postinfection (Fig. 3C). To confirm our results, we further studied the ASFV-DP distribution compared to that of the ASFV major capsid protein p72, which localizes to the viral factories (34, 35). At 16 hpi, p72 was found within the viral factories, while ASFV-DP was located mainly in the surroundings of these structures (Fig. 3D).

### ASFV-DP interacts with cellular ribosome components during ASFV infection.

**FIG 4:**
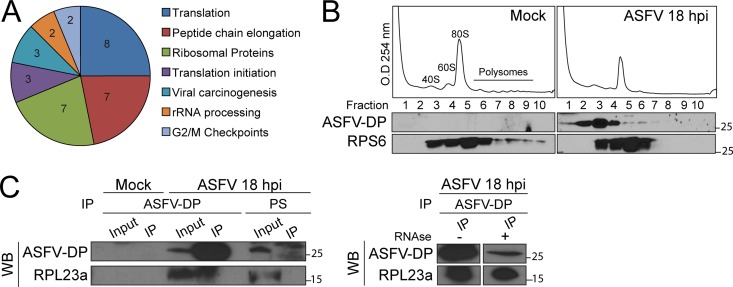
ASFV-DP interacts with the cellular translation machinery. (A) Analysis of the proteins enriched in the GST–ASFV-DP fraction after incubation with a sample from 18 hpi with ASFV. The numbers in the segments represent the quantities of proteins found in each category. (B) ASFV-DP cosediments with the ribosome. Mock- And 18-hpi infected samples were subjected to polysome profile analysis, fractions were recovered, and ASFV-DP and RPS6 presence was determined by Western blotting. (C) Analysis of the interaction of ASFV-DP and ribosomal protein RPL23a. Vero cells were infected with ASFV (MOI = 5 PFU/cell) and harvested at 18 hpi. Samples were incubated with anti-ASFV-DP or PS, and ASFV-DP and RPL23a presence was revealed by Western blotting (WB). The presence (+) or absence (−) of RNase A/T1 treatment is indicated.

To identify the interacting partners of ASFV-DP, we performed pulldown assays using glutathione *S*-transferase (GST)–ASFV-DP incubated with extracts from mock- or ASFV-infected Vero cells at 6 and 18 hpi, followed by proteomic analysis (see Table S1 in the supplemental material). Unfused GST was used to discriminate between specific and nonspecific interactions. We observed that under all the conditions tested (mock infection, 6 hpi, and 18 hpi), cytoplasmic ribosome proteins from the small (RPS6, RPS14, and RPS3) and large (RPL23a, RPL24, and RPL8) subunits were enriched in the ASFV-DP fraction (Fig. 4A; see Fig. S1A and Tables S1 and S2 in the supplemental material). In order to validate this result, cosedimentation experiments were performed. Briefly, mock- and ASFV-infected cells were subjected to polysome profile analysis, the different fractions were recovered, and ASFV-DP and RPS6 presence was analyzed by Western blotting. Anti-RPS6 was used to detect the ribosome. As shown in Fig. 4B, ASFV-DP cosedimented with this complex and was mainly detected with the 40S subunit (suggesting a role in the initiation translation complex and mRNA) but was also present in the 60S, 80S, and polysome fractions. Interestingly, immunoprecipitation (IP) followed by Western blotting confirmed that ASFV-DP interacts with the ribosomal protein RPL23a in infected cells (Fig. 4C). This interaction was specific and RNA independent, since (i) no interaction between ASFV-DP and other translation factors, such as PABP, RPS6, or RPL24, appeared to occur in living cells (see Fig. S1B in the supplemental material), and (ii) treatment with RNAses A/T1 did not affect the interaction (Fig. 4C, right). It is noteworthy that this is the first time that an interaction between the ribosome and an ASFV protein has been described.

### ASFV-DP colocalizes with the cap structure during infection.

**FIG 5:**
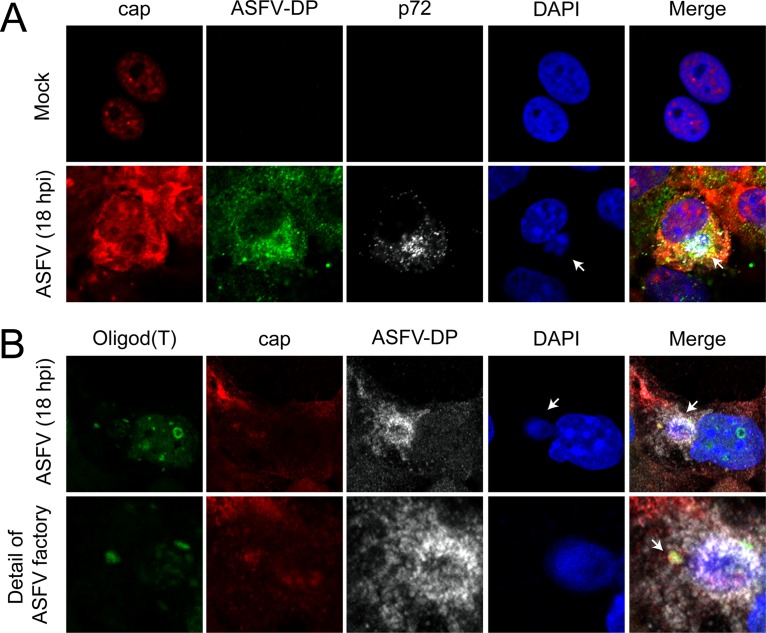
ASFV-DP colocalizes with the cap structure. COS-7 cells were infected with ASFV (MOI = 5 PFU/cell) and fixed at 18 hpi. (A) Localization of mRNA cap structures in ASFV-infected cells. Cap (red), ASFV-DP (green), and p72 (gray) were detected by using an anti-cap antibody, the anti-ASFV-DP serum, and an anti-p72 antibody, respectively. Cellular and viral DNA were stained with DAPI (blue); the arrows indicate viral factories. (B) Localization of poly(A) RNA in ASFV-infected cells. Poly(A) RNA (green), cap (red), and ASFV-DP (gray) were detected by using a fluoresceinated oligo(dT) probe, the anti-cap antibody, and the anti-ASFV-DP serum, respectively. Cellular and viral DNAs were stained with DAPI (blue); the arrows indicate the colocalization of poly(A) RNA, the cap structure, and ASFV-DP.

As the cap structure is a necessary part of the decapping process, its distribution was examined in the context of ASFV infection to assess a possible relationship with ASFV-DP. A specific antibody able to recognize both m^7^G and m_3_G structures (red) (36) was used, the nuclei were stained with DAPI (blue), and viral factories were localized using the specific anti-p72 antibody (gray), while ASFV-DP (green) was detected using our specific antiserum (Fig. 5A). In mock-infected cells, the cap structure was strongly localized in the nucleus and more diffusely in the cytoplasm (Fig. 5A, top row), in agreement with the fact that capped RNAs are present in both compartments. Interestingly, the cap signal in ASFV-infected cells was clearly increased in the cytoplasm, especially at the peripheries of viral factories, where it colocalized with ASFV-DP (Fig. 5A, bottom row). The observed decrease in the cap signals in the nuclei of infected cells may have been due to impairment of transcription, which is a common phenomenon during infection by cytolytic viruses (37–41). Importantly, the increased cap signal at the viral replication sites may correspond to capped viral mRNAs, which have previously been described surrounding these structures (26). In order to validate this hypothesis, we performed fluorescent *in situ* hybridization (FISH) experiments to detect the locations of the poly(A) RNA, the cap structure, and ASFV-DP. COS-7 cells were infected with ASFV and a fluoresceinated oligo(dT) probe, the anti-cap antibody, and the anti-ASFV-DP antibody were added to identify both viral and cellular mRNAs, the cap structure, and the decapping protein, respectively (Fig. 5B). We observed that poly(A) mRNA was clearly detected at the viral factories in infected cells, where it colocalized with the cap structure and with ASFV-DP. No oligo(dT) signal was observed to be spread in the cytoplasm of infected cells, suggesting that the RNA detected might be viral mRNA. This result is consistent with the previous data and indicates that the cap signal observed in Fig. 5A was due to both capped mRNAs and liberated cap structures produced after the decapping activity of ASFV-DP, since not all the cap signal colocalized with the oligo(dT) signal (Fig. 5B). The fact that the capped RNAs colocalized with ASFV-DP supports the role of ASFV-DP as an *in vivo* decapping enzyme. It is noteworthy that the overall cap signal intensity was slightly but reproducibly enhanced in ASFV-infected cells compared to uninfected cells (see Fig. S1C in the supplemental material). ASFV infection caused a decrease in host mRNA levels as measured by RT-qPCR (Fig. 1B) and oligo(dT) hybridization (26); however, we did not observe such a decline in cap levels, likely due to the fact that the antibody used recognized both mRNA and free cap.

### ASFV-DP binds cellular poly(A) RNA in cultured cells.

**FIG 6:**
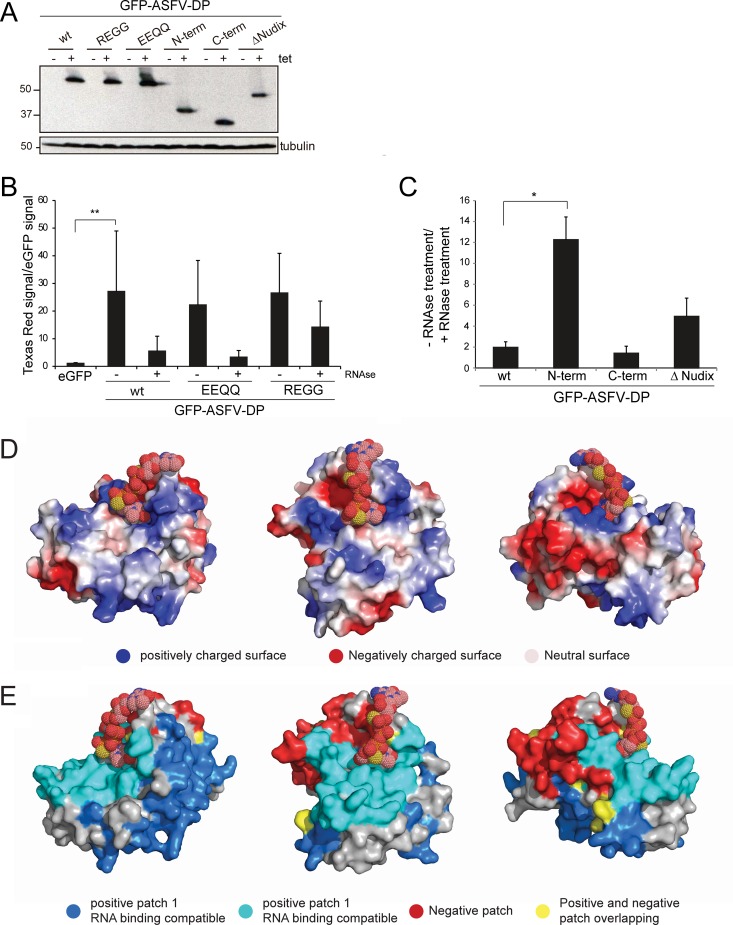
ASFV-DP binds poly(A) RNA in cultured HeLa cell lines. (A) Size analysis of recombinant GFP–ASFV-DP proteins by Western blotting. A specific anti-GFP antibody was used to detect expression of GFP–ASFV-DP fusion proteins; tubulin was used as a loading control. Numbers on the left are kilodaltons. (B) HeLa cells were induced with tetracycline for expression of eGFP, GFP–ASFV-DP wt, and GFP–ASFV-DP catalytic mutants (REGG and EEQQ). The levels of RNA bound to the different fusion proteins were determined by the dual-fluorescence method. Treatment with RNases A and T1 was carried out as a specificity control; The presence (+) or absence (−) of this treatment is indicated. RNA values (means and SD) are expressed as the ratio of Texas Red signal to eGFP signal relative to eGFP. **, *P* < 0.01 (*n* = 3). (C) Levels of RNA bound to the full-length GFP–ASFV-DP wt and truncated mutants GFP–ASFV-DP N-term, GFP–ASFV-DP C-term, and GFP–ASFV-DP ΔNudix were measured using the dual-fluorescence method. RNA values (means and SD) are expressed as the ratio of the RNase treatment to no RNase treatment relative to eGFP. *, *P* < 0.05 (*n* = 3). (D) Electrostatic surface potential of ASFV-DP. The protein surface is represented according to charge. (E) BindUP RNA-binding surface prediction. The most likely RNA-binding surfaces are represented in cyan and blue, while the nonbinding patch is represented in red. Overlapping regions are represented in yellow. Cap structure was modeled by using a sphere representation.

As decapping enzymes must bind mRNA to catalyze the decapping reaction, the RNA-binding activity of ASFV-DP was tested. Six stable HeLa Flip-TREx cell lines were generated that expressed green fluorescent protein (GFP)-tagged ASFV-DP variants: GFP–ASFV-DP wild type (wt), GFP–ASFV-DP REGG, GFP–ASFV-DP EEQQ, GFP–ASFV-DP N-term, GFP–ASFV-DP C-term, and GFP–ASFV-DP ΔNudix. The expression of the different fusion proteins in HeLa Flip-TREx cells was studied after induction in the presence of tetracycline (Tet) (Fig. 6A).

To analyze RNA binding of each recombinant protein, a dual-fluorescence method was used, as previously described (42–44). Briefly, the different cell lines were incubated with 4-thiouridine (4-SU) 2 h prior to induction with Tet and incubation overnight (18 h). As 4-SU is incorporated into nascent RNA and protein-RNA cross-links after irradiation with 365-nm UV light, this method was found to be the most efficient for this study. GFP–ASFV-DP associated with RNA at an ∼25-fold-higher level than the negative control, enhanced GFP (eGFP) (Fig. 6B). In addition, the ASFV-DP catalytic mutants REGG and EEQQ bound to RNA to an extent similar to that of the wt protein, suggesting that these mutations, although important for catalytic activity *in vitro* (28), are likely not involved in RNA binding.

Importantly, when we analyzed the RNA binding to the truncated ASFV-DP proteins, we observed that the N-terminal fragment of ASFV-DP was able to bind ∼10-fold more RNA than the C terminus or the full-length protein. The ΔNudix variant showed an intermediate phenotype, binding more RNA than the wt but less than the N-terminal (N-term) fragment (Fig. 6C). Taken together, these results point to the ASFV-DP N terminus as the major mediator of RNA interaction. On the other hand, the Nudix motif, though indispensable for decapping activity *in vitro* (20, 28, 45, 46), does not seem to be essential for RNA binding. Interestingly, the absence of the Nudix motif seems to contribute to stability of RNA binding, possibly due to the lack of decapping activity, and consequently the lack of RNA degradation, expected in this variant.

To predict the most likely RNA-binding surface within ASFV-DP, we used BindUP software (Israel Institute of Technology), which calculates the local electrostatic potential to predict protein surfaces with potential to bind RNA (Fig. 6D and E). BindUP identified two large positively charged patches in ASFV-DP as candidates for mediating the interaction with RNA (Fig. 6E, blue and cyan). The amino acids forming these regions are listed in Table 2. In agreement with the previous data, the cyan positively charged surface overlaps the N-terminal region of the protein that exhibits RNA-binding activity (Fig. 6C). In contrast, most of the nonbinding, negatively charged patch coincides with the C-terminal moiety (Fig. 6E, red).

**TABLE 2:** Amino acids present in the electrostatic patches at the surface of ASFV-DP

Name	Region	Charge	Amino acids
Patch 1	N terminal	Positive	ILE5 CYS7 ARG10 MET11 ASN12 THR13 GLN17 SER32 PHE34 PHE52 ARG60 LEU61 LEU62 VAL63 LYS64 THR65 LEU66 ASP67 ASP69 ARG70 TYR73 HIS74 TYR93 HIS94 LYS95 LYS96 TYR97 GLN98 LYS99 PHE100 LYS108 ASN109 ASP114 ASN117 LYS119 LYS120 LEU121 ILE122 SER123 LEU134 LEU135 TRP136 GLU137 ILE138 ILE163 THR164 TYR168 LYS176 MET178 TYR180 HIS189 TYR191 PHE192 MET195 LEU196 CYS197 LYS198 ASN206 LEU207 SER208 LEU209 SER220 LYS221 ILE222 SER223 TRP224 GLN225 ASN226 MET227 GLU228 ALA229 VAL230 ARG231 PHE232 ILE233 SER234 LYS235 ARG236 GLN237 SER238 PHE239 ASN240 GLU242 PRO243 ILE245 ALA248 ASN250 PHE251 ILE252 LYS253 ASN254 TYR255 LEU256 ARG257 TYR258 LYS259 HIS260
Patch 2	N terminal	Positive	ILE21 LEU22 LYS25 ARG26 TYR27 SER28 LEU29 SER32 GLU33 PHE34 ILE35 HIS36 ILE21 LEU22 LYS25 ARG26 TYR27 SER28 LEU29 SER32 GLU33 PHE34 ILE35 HIS36 LYS140 GLY141 LYS142 PRO143 LYS144 ARG155 GLU156 GLU160 ASP182 LYS184 TYR187 LYS188 MET244 ILE245 PRO247
Patch 3	Nudix motif and C terminal	Negative	LYS99 PHE100 ARG101 LYS108 ASN109 LEU124 ASN126 SER131 GLU137 LYS140 GLU145 ASP146 GLU147 SER148 ASP149 LEU150 THR151 ILE154 ARG155 GLU156 GLU158 GLU159 GLU160 GLY162 THR164 GLU166 TYR167 LEU171 GLU173 THR185 GLU186 HIS189 LEU209 GLN210 GLU212 ARG215 GLU218

Since both ASFV-DP and Dcp2 are largely conserved, we used the Dcp1-Dcp2-PNRC2-cap analog complex (Protein Data Bank [PDB] accession no. 5KQ4) (47) as a reference to position the cap in the ASFV-DP structure (Fig. 6D and E). Interestingly, the cap structure interacts at the interface between the basic (cyan) and acidic (red) patches. The acidic surface contacting the cap is composed of an α-helix rich in glutamic acid residues that actually corresponds to the Nudix domain (Fig. 6D, right). These results agree with data previously reported for other decapping enzymes, conferring importance on the N-terminal region in the interaction with RNA (48, 49).

**FIG 7:**
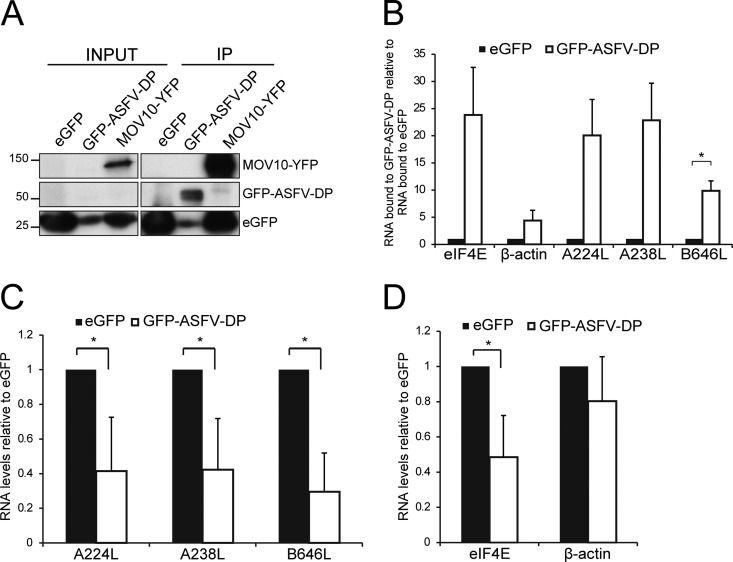
GFP–ASFV-DP is able to bind both viral and cellular mRNAs in the context of ASFV infection, decreasing both types of mRNA. (A) After induction of the recombinant proteins with Tet, HeLa cells were infected with ASFV (MOI = 5 PFU/cell) and cross-linked (cCL) at 7 or 14 hpi. eGFP and GFP–ASFV-DP wt were immunoprecipitated with GFP-Trap_A beads, and bound RNA was extracted using phenol-chloroform-isoamyl alcohol and analyzed by RT-qPCR. HeLa cells expressing MOV10-YFP were used as positive controls, and immunoprecipitation was confirmed by anti-GFP Western blotting prior to RNA extraction. (B) Quantification of representative cellular (eIF4E and β-actin) and viral (A224L, A238L, and B646L) mRNAs extracted from immunoprecipitated fusion proteins by RT-qPCR at 14 hpi. The values are normalized to the RNA input (means and SEM; *n* = 3). *, *P* < 0.05. (C and D) Viral A224L, A238L, and B646L (C) and cellular eIF4E and β-actin (D) mRNA levels were analyzed by RT-qPCR using specific primers after GFP–ASFV-DP induction. HeLa cells were induced to express eGFP or GFP–ASFV-DP wt 18 h prior to ASFV infection (MOI = 5 PFU/cell). At 8 hpi, cells were harvested and total mRNA was extracted; the values are normalized to 18S RNA (medians ± SD; *n* = 3). *, *P* < 0.05.

Next, the ability of ASFV-DP to bind poly(A) RNA in the context of the infection was studied. HeLa Flip-TREx cells were induced to stably express eGFP and GFP–ASFV-DP wt and subsequently infected with ASFV for 14 h. RNA-protein interactions were immobilized by conventional cross-linking (cCL) (254-nm UV irradiation), as 4-thiouridine resulted in decreased expression of some viral proteins (see Fig. S2 in the supplemental material). GFP complexes were immunoprecipitated with GFP Trap_A beads (see below) and checked for quality by Western blotting using an anti-GFP antibody. MOV10-yellow fluorescent protein (YFP)-expressing cells were used as a positive control due to its well-known property as an RNA-binding protein, while unfused eGFP was used as a negative control (Fig. 7A) (42). RNA in the GFP_Trap eluates was analyzed by RT-qPCR using oligonucleotides against representative cellular (eIF4E and β-actin) and viral (A238L, A224L, and B646L) mRNAs. Interestingly, GFP–ASFV-DP was able to bind both viral and cellular mRNAs in the context of ASFV infection, in contrast to the negative control, eGFP (Fig. 7B). Interestingly, the RNA-binding activity of ASFV-DP presented a degree of specificity, as mRNA from β-actin associated to a lesser extent than eIF4E mRNA (Fig. 7B). Since ASFV-DP selectively interacted with both cellular and viral RNAs, we hypothesized that it might be involved in both cellular shutoff and temporal regulation of viral protein expression.

### ASFV-DP overexpression reduces cellular and viral mRNAs.

In order to further study the effect of ASFV-DP on the stability of viral and cellular mRNAs, representative viral (A238L, A224L, and B646L) and cellular (eIF4E and β-actin) mRNA levels were tested by RT-qPCR in mock- and ASFV-infected HeLa Flip-TREx cells. In HeLa cells overexpressing GFP–ASFV-DP, all viral mRNAs were decreased compared to eGFP control cells (Fig. 7C). Levels of cellular eIF4E mRNA were also decreased in ASFV-infected HeLa cells expressing GFP–ASFV-DP (Fig. 7D). This result indicates that ASFV-DP is able not only to degrade viral mRNA, but also to decrease cellular mRNA in the context of infection. Moreover, the degradation appears to be selective, as the ASFV-DP effect is more evident on eIF4E mRNA than on β-actin mRNA.

In summary, these results point to ASFV-DP as a new regulator of viral and host mRNA stability in infected cells.

## DISCUSSION

Due to the limited size of their genomes, viruses rely on the host translation machinery for efficient synthesis of their proteins (50). Therefore, viruses must manipulate cellular mRNA metabolism and translation, impairing host protein synthesis and liberating cellular resources in order to ensure and facilitate their own replication (4, 51). Our previous results demonstrated that ASFV causes a progressive decrease in poly(A) mRNA signal in the cytoplasm of infected cells, which is detectable at the peripheries of viral factories at late times postinfection (26). In this work, we found that ASFV triggers general degradation of cellular mRNA, as all cellular genes tested by RT-qPCR decreased after infection (Fig. 1B), concomitant with reduced cellular protein synthesis (25–27). The global degradation of host mRNAs should thus contribute to the host shutoff. General inhibition of host protein synthesis, or cellular shutoff, is accompanied by a simultaneous increase in viral protein synthesis (27). Conversely, the persistency of viral mRNA translation under these conditions implies that viral mRNA should be refractory (at least partially) to such degradation. Additionally, ASFV is known to exercise temporal control over the expression of its genes (24), a process that inherently involves regulation of both mRNA transcription and degradation.

ASFV-DP was found to harbor a functional Nudix motif similar to those found in cellular and viral decapping enzymes, including D9 and D10 of VACV (Fig. 2A). Due to their different expression times during infection, it has been suggested that D9 and D10 act in overlapping and complementary manners (19, 52). However, as ASFV-DP is the only enzyme in the ASFV genome that possesses a Nudix motif, it is hypothesized that it has a unique decapping function during infection, being responsible for both cellular shutoff and temporal control of viral gene expression, further supported by its constitutive expression (Fig. 3A and B).

ASFV-DP was found to localize to the ER (Fig. 3C), as was previously suggested by Cartwright et al. (29). The authors proposed that, since the ASFV-DP sequence does not harbor any ER peptide signal, it probably associates with the ER on the cytosolic side without translocating into the ER lumen. They also suggested that this localization may be related to a specific activity of the protein. In this study, cosedimentation experiments demonstrated that ASFV-DP is present in different ribosomal fractions. Moreover, GST pulldown and coimmunoprecipitation assays revealed that ASFV-DP is able to interact with the ribosomal protein L23a (Fig. 4C; see Table S1 in the supplemental material). These data support ASFV-DP presence at the ER and translation complexes. Interestingly, it has been reported that other viral proteins implicated in mRNA regulation, such as the SOX endonuclease of Kaposi's sarcoma-associated herpesvirus (KSHV), also cosediment with the 40S ribosomal subunit (14). It was suggested that SOX may be recruited to the mRNA by association with translation initiation machinery; it may be possible that ASFV uses a similar mechanism to facilitate decapping activity on translationally active host mRNAs. Moreover, the presence of ASFV-DP at the viral factories could correlate with the function of the protein as a regulator of viral mRNA stability (Fig. 3C and D).

In order to determine the relationship between mRNA, the cap structure, and ASFV-DP, we used both an oligo(dT) probe and a specific antibody that recognizes all m^7^G and m_3_G structures, including capped mRNAs. In mock-infected cells, cap signal is spread through the cell and accumulates in the nucleus. However, in ASFV-infected cells, cap-derived nuclear signal decreases, correlating with an increase in the cytoplasm. Interestingly, both poly(A) RNA and the cap signal partially colocalize with ASFV-DP at the peripheries of the viral factories at late times after infection (Fig. 5A and B). Viral mRNAs are present at the replication sites in VACV (53), while D10 is excluded from the viral factories (52). Hence, factory exclusion has been proposed as a strategy to avoid degradation of late viral mRNAs (18).

It has been reported that ASFV-DP has two enzymatic activities *in vitro*. The first functional description of the protein demonstrated that it possesses a high affinity for PP-InsP5 (diphosphoinositol polyphosphate), implying a role in viral membrane morphogenesis (29). However, a second study demonstrated that it was able to hydrolyze cap structures when attached to an RNA body, a common feature of decapping enzymes (18, 28, 54). In this work, we showed for the first time that ASFV-DP is able to bind RNA in cultured cells (Fig. 6B). Though substitutions for conserved glutamates of the Nudix motif impair decapping activity (28), we demonstrated that these mutations do not affect RNA binding (Fig. 6B). Given that the highly conserved residues are catalytically important in the Nudix motif of SpDcp2 (55), it can be hypothesized that glutamates could play a similar role in the ASFV-DP catalytic pocket.

We further identified the N terminus as sufficient to mediate ASFV-DP interaction with RNA, whereas the C-terminal region did not seem to be involved (Fig. 6C). Electrostatic surface analysis supported these results, revealing that the N terminus possesses a basic channel that may serve as an RNA-binding platform (Fig. 6D and E). A positively charged channel is also present on the dorsal surface of the Nudix regions of S. pombe, S. cerevisiae, and human Dcp2 enzymes, and mutations of residues in this channel have been shown to decrease Dcp2 substrate affinity (48, 49, 56). However, the amino acids of this region are not clearly conserved in D10 and ASFV-DP (18, 57). Unlike the cap-binding cavity, basic amino acids of the positive channel are only loosely conserved at the primary-sequence level between SpDcp2 and ScDcp2 (18, 48). This suggests that the positive charge of the surface is the most important signature for RNA binding. Interestingly, when the modeling of the protein was done, we realized that the predicted Nudix motif and the C terminus of ASFV-DP are more similar to Dcp2 structure than the N terminus. ASFV-DP may possess an N-terminal surface with optimized RNA-binding characteristics compared to Dcp2. A reason could be that ASFV-DP function is not complemented by other proteins (as occurs with Dcp1), making ASFV-DP the only viral protein responsible for mRNA decapping during ASFV infection.

Our results show that the N-terminal fragment's RNA-binding activity is far superior to that of the full-length ASFV-DP. The N-terminal domain thus suffices to bind RNA, suggesting that the Nudix motif, and thus ASFV-DP catalytic activity, is not required for interaction with RNA. However, ASFV-DP mediates the hydrolysis of the cap structure, and such a reaction should involve a transitory interaction with the RNA to allow RNA release after decapping. Interestingly, the ASFV-DP construct lacking the Nudix motif is also able to bind RNA, although to a lesser extent than the N-terminal fragment alone, suggesting that it is not the catalytic activity *per se* that determines the lower RNA-binding avidity of ASFV-DP (Fig. 6C). A plausible mechanistic explanation is that the interplay of the positively charged N-terminal domain with the highly acidic C terminus could increase the disassociation (off) rate in the RNA–ASFV-DP complex (Fig. 6D). This is a likely explanation, since the acidic patch generated by the C terminus domain is in close proximity to the putative RNA-binding surface. Because the repulsion effect of the C terminus domain is lost in the N-terminal construct, it would likely increase the duration or the strength of the interaction with RNA (Fig. 6C). In agreement with our results, *in vitro* studies with human Dcp2 to identify the minimal region with decapping activity showed that a construct harboring an incomplete Nudix fold (and thus lacking enzymatic activity) was able to bind RNA (56). Further mutational studies affecting different regions of the ASFV-DP N-terminal region must be carried out to determine the precise amino acids implicated in RNA binding.

We have established that ASFV-DP not only binds cellular mRNA in cultured cells, but also binds host and viral mRNAs in the context of infection (Fig. 7B). The fact that ASFV-DP interacts with cellular mRNAs suggests that it is involved in cellular shutoff, while its interaction with viral mRNAs may be related to temporal viral gene regulation. This hypothesis is also supported by the fact that when ectopically overexpressed, ASFV-DP causes a decrease of both cellular and viral mRNA levels (Fig. 7C and D). Additionally, ASFV-DP could interact with viral mRNA in order to avoid accumulation of double-stranded RNA (dsRNA) and the cellular immune response, as has been described for D9 and D10 during VACV infection (58). ASFV-DP showed higher binding affinity for eIF4E mRNA than for actin mRNA (Fig. 7B), suggesting that it displays specific affinities for determinate cellular substrates. Selectivity in host mRNA degradation has been previously described in other viral systems, such as alphaherpesvirus (59) and VACV (60), whose D9, which presents higher affinity for the RNA body than D10, may be able to differentiate mRNA based on sequences (19). In a similar manner, ASFV may discriminate among cellular transcripts, using ASFV-DP as a tool to target specific cellular mRNAs. ASFV-DP has been shown to be more strongly inhibited by uncapped mRNAs than D9, and it is not affected by methylated cap analogs (28). Additionally, *in vitro* RNA-binding assays demonstrated that while ASFV-DP was able to interact with uncapped cellular mRNAs, the observed shift in electrophoretic mobility shift assays (EMSAs) was not complete (28). The authors suggest two explanations: the requirement for the cap structure to stabilize ASFV-DP–RNA interaction and the fact that ASFV-DP displays strong affinity only for molecules with certain features. These explanations fit our data and are also in agreement with what has been described for Dcp2 (61, 62).

Remarkably, lower levels of p72 mRNA were bound by ASFV-DP than by A224L and A238L (Fig. 7B), supporting the possibility that ASFV-DP displays selectivity among early and late viral mRNAs. Viral mRNA specificity has also been suggested for D10 of VACV, with a stronger affinity for the m^7^GpppG cap analog structure (20). Whether ASFV-DP displays some degree of sequence selectivity deserves further consideration.

In conclusion, the results of this study indicate that ASFV-DP is an early viral protein that is also detected at late times, which presents different subcellular locations during infection: at early times, it mainly localizes to the ER, while at later times, it accumulates at the viral factories, where it partially colocalizes with the cap structures and interacts with ribosomes via binding to RPL13a. ASFV-DP is the first ASFV protein reported to display this ability. Additionally, ASFV-DP is an RNA-binding protein able to interact with both cellular and viral mRNAs in the context of infection, which led to a decrease of both cellular and viral mRNAs when overexpressed. Taken together, these results indicate that ASFV-DP acts as a viral regulator of both cellular and viral mRNA metabolism, impairing cellular protein synthesis and ensuring viral temporal gene expression.

## MATERIALS AND METHODS

### Cell culture and virus strains.

Vero (ATCC CCL 81), COS-7 (ATCC CLR 1650) (epithelial and fibroblast kidney cells from African green monkeys, respectively), and HeLa (ATCC CCL-2; epithelial cells of the human cervix) cells were obtained from the American Type Culture Collection. HeLa GFP stable cell lines were obtained, as detailed below, from the HeLa cell line. All three cell types were cultivated in Dulbecco's modified Eagle's medium (DMEM) (63) supplemented with 5% fetal bovine serum (FBS) and maintained at 37°C in a controlled 7% CO_2_ atmosphere saturated with water vapor. The growth medium was supplemented with 2 mM l-glutamine, 0.4 mM nonessential amino acids, and 0.05 mg/ml gentamicin for Vero and COS-7 cells and 0.1 mg/ml penicillin plus 10^2^ U streptavidin for HeLa cells. Induction of protein expression in stable HeLa cell lines was performed by addition of 1/4,000 Tet (5 mg/ml) for 16 to 20 h.

Infections were carried out with the Vero-adapted isolate of ASFV strain Ba71 (Ba71V) and titrated by plaque assay in Vero cells, as described previously (64). HeLa and Vero cell infections were performed in DMEM with 2% FBS, while COS-7 cells were infected in DMEM with 5% FBS. Depending on the requirements of the experiment, an MOI from 1 to 5 was used.

### Plasmid construction, mutagenesis, and generation of stable HeLa cell lines.

For generation of the ASFV-DP–HA vector, we cloned the D250R gene (strain Ba71V), together with the 250-bp upstream fragment, into a pcDNA vector containing an HA tag (a kind gift from Ricardo Madrid, Bioassays, Parque Científico de Madrid, Madrid, Spain). The D250R gene was amplified using PCR primers KpnI_promD250R_FW and BamHI_noSTOP_D250R_RV (Table 3) to construct a D250R gene with an N-terminal HA fusion, with expression of the protein under the control of the viral promoter.

**TABLE 3:** Primers used in this work

Name	Sequence
KpnI_promD250R_FW	GCGGGTACCTGAATATCTTGTGAACACAGC
BamHI_noSTOP_D250R_RV	GCGGGATCCGTGCTTATATCGTAAATAGTTTTTAATAAA
BamHI_g5Rwt_FW	GGGGGATCCATGGATACTGCCATGCAGCTT
EcoRI_g5Rwt_RV	GGGGAATTCCTACTAGTGCTTATATCGTAA
EcoRI_D250R_FW	GGGGAATTCATGGATACTGCCATGCAGCTT
BamHI_D250R_RV	GGGGGGGGATCCCTACTAGTGCTTATATCGTAA
D250R_mutREGG_FW	TCGGACCTTACCTGTGCCATAGGGGGGTTTGAAGAAGAAACCGGGATT
D250R_mutREGG_RV	AATCCCGGTTTCTTCTTCAAACCCCCCTATGGCACAGGTAAGGTCCGA
D250R_mutEEQQ_FW	TGTGCCATACGGGAGTTTGAACAACAAACCGGGATTACCCGCGAATAT
D250R_mutEEQQ_RV	ATATTCGCGGGTAATCCCGGTTTGTTGTTCAAACTCCCGTATGGCACA
KpnI_GFP_FW	GGGGGTAACATGGTGAGCAAGGGCGAGGAG
Nexo_GFPpD250R_FW	TCGAGCTCAAGCTTCGAATTCATGGATACTGCCATGCAGCTT
Nexo_GFPpD250R_RV	AAGCTGCATGGCAGTATCCATGAATTCGAAGCTTGAGCTCGA
BamHI_pD250R_RV	GGGGGGGGATCCCTACTAGTGCTTATATCGTAA
BamHI_pD250R_N-term_RV	GGGGGATCCCTACTAAATTTCCCATAGAAGTGT
Nexo_GFP_pD250R_Cterm_FW	AGCTCAAGCTTCGAATTCACCCGCGAATATTACCAG
Nexo_GFP_pD250R_Cterm_RV	AATCTGGTAATATTCGCGGGTGAATTCGAAGCTTGAGCTCGA
pD250RNtermCterm_FW	TCAGAAACACTTCTATGGGAAATTACCCGCGAATATTACCAGATTCTC
pD250RNtermCterm_RV	AATCTGGTAATATTCGCGGGTAATTTCCCATAGAAGTGTTTCC
18S FW	GCAATTATTCCCCATGAACG
18S RV	GGGACTTAATCAACGCAAGC
β-actin FW	AGGTCATCACCATTGGCAAC
β-actin RV	CGTGGATGCCACAGGACT
BIRC2 FW	GCTGACCCACCAATTATTCATTTTGG
BIRC2 RV	CACACACGTCAACTGTTGAAAGAGAGC
eIF4A1 FW	AATCCGCATCTTGGTGAAAC
eIF4A1 RV	CCCTCTCCACTGCCACAA
eIF4E FW	CAGCAGAGACGAAGTGACCTT
eIF4E RV	ACATTAACAACAGCGCCACA
p53 FW	GGCCCATCCTTACCATTATCA
p53 RV	AGCTGTTCCGTCCCAGTAGA
A238L FW	GCAGATCCGACTCAAAAAGACT
A238L RV	ACTCCATATTTCCTGTAAAGACTGC
A224L FW	AAGCACCCTTTACAGGTGCATGGC
A224L RV	TCCTATAATGCCCAAGGTTGCACGG
B646L FW	CCCGAGAACTCTCACAATATCC
B646L RV	CGTTGCGTCCGTAATAGGAG

GST–ASFV-DP was produced for use in generation of specific anti-ASFV-DP serum and pulldown assays by cloning the g5R gene (the Malawi Lil 20/1 virus strain) from pCDNAg5R (a kind gift from L. Dixon, The Pirbright Institute, Pirbright, United Kingdom) into pGEX-2T using PCR primers BamHI_g5Rwt_FW and EcoRI_g5Rwt_RV (Table 3). The resultant pGEX-ASFV-DP was transformed in Escherichia coli (BL21) to produce GST–ASFV-DP proteins under the conditions described below.

For study of the catalytic activity of ASFV-DP, we performed substitutions affecting two conserved amino acids within the Nudix motif. In the REGG mutant, arginine 146 and glutamate 147 were replaced by glycine (R146G/E147G), whereas in the EEQQ mutant, glutamates 150 and 151 were replaced by two glutamines (E150Q/E151Q). To generate these mutants, the D250R gene was first cloned into the pCFP-C2 vector to generate pCFP-ASFV-DP wt. Cloned fragments were amplified by PCR from a lysate of cells infected by ASFV Ba71V using primers EcoRI_D250R_FW and BamHI_D250R_RV (Table 3). Construction of pCFP ASFV-DP REGG and pCFP ASFV-DP EEQQ was carried out by PCR, using the pCFP-ASFV-DP wt vector as a template and primers D250R_mutREGG_FW and D250R_mutREGG_RV and D250R_mutEEQQ_FW and D250R_mutEEQQ_RV, respectively (Table 3).

Inducible HeLa Flp-in TREx cell lines were established for the expression of GFP-tagged ASFV-DP wt and REGG/EEQQ mutants. pEGFP-C2, pCFP-ASFV-DP wt, and the pCFP-ASFV-DP REGG/EEQQ constructions were used as DNA templates to clone into the pCDNA5/FRT/TO vector using primers KpnI_GFP_FW, nexo_GFPpD250R_FW, nexo_GFPpD250R_RV, and BamHI_pD250R_RV (Table 3). pCDNA5/FRT/TO vectors containing ASFV-DP wt and mutants were transfected into HeLa Flp-in TREx cell lines, generated as described previously (42, 43). Positive cells were enriched by fluorescence-activated cell sorting (FACS), which was done in the Flow Cytometry Core Facility of the European Molecular Biology Laboratory, Heidelberg, Germany.

Three additional HeLa Flp-in TREx cell lines were produced: HeLa GFP–ASFV-DP N-term (GFP fused to the D250R N terminus), HeLa GFP–ASFV-DP C-term (GFP fused to the D250R C terminus), and HeLa GFP–ASFV-DP ΔNudix (Nudix deletion). We stablished the regions by taking the Nudix motif as a reference: the N-terminal region contains the sequence of ASFV-DP upstream of the Nudix conserved motif, while the C-terminal region of the protein covers the sequence downstream from the Nudix sequence. Finally, GFP–ASFV-DP ΔNudix possesses both N-terminal and C-terminal regions but lacks the ASFV-DP Nudix motif. GFP–ASFV-DP N-term was produced by fusion PCR using primers KpnI_GFP_FW, nexo GFP_pD250R_FW, nexo_GFPpD250R_RV, and BamHI_pD250R_N-term_RV (Table 3) and using pEGFP-C2 and pCFP-pD250R wt as DNA templates. HeLa GFP–ASFV-DP C-term was obtained by fusion PCR using KpnI GFP FW, nexo_GFP_pD250R_Cterm_FW, nexo_GFP_pD250R_Cterm_RV, and BamHI_pD250R_RV primers (Table 3), using pEGFP-C2 and pCFP-ASFV-DP wt as DNA templates. HeLa GFP–ASFV-DP ΔNudix was obtained by two fusion PCRs, using the primers KpnI_GFP_FW, nexo_GFP_pD250R_FW and RV, pD250RNtermCterm_FW and pD250RNtermCterm_RV and BamHI_pD250R_RV (Table 3), with pEGFP-C2 and pCFP-pD250R wt used as DNA templates.

In all cases, restriction digestions with the specific enzymes were performed following the manufacturer′s instructions (NEB). HeLa eGFP and HeLa MOV10-YFP stable cell lines were used as negative and positive controls for RNA-protein binding assays (43).

### Generation of ASFV-DP-specific antisera.

A GST fusion protein was prepared for the g5R gene (Malawi Lil 20/1) in E. coli BL21 cells transformed with the pGEX-ASFV-DP vector and grown under low-oxygen conditions overnight at 37°C. After 16 h, samples were recovered and lysed in lysis buffer (50 mM Tris HCl, pH 7.5, 0.5 M ClNa, 1 mM EDTA, 7 mM β-2-mercatptoethanol, 5% glycerol). GST–ASFV-DP was purified using glutathione columns (Sigma) and eluted with eluting buffer (50 mM Tris HCl, pH 8, 100 mM ClNa, 10 mM glutathione). Correct expression, solubility, and purity of the wt GST–ASFV-DP was analyzed by Coomassie blue staining and Western blotting using an anti-GST antibody. The purified GST–ASFV-DP protein was used to generate specific antibodies in two rabbits by subcutaneous injection of 500 μg of GST–ASFV-DP (per rabbit) with 50% complete Freund's adjuvant (Sigma). Fifteen days after the first immunization, the rabbits were subcutaneously boosted with 250 μg of GST–ASFV-DP and 50% complete Freund′s adjuvant. Successive monthly intramuscular inoculations (250 μg of protein and 50% incomplete Freund′s adjuvant) were performed for 6 months, with serum samples collected from both animals after the third, fifth, and seventh boosts and tested by Western blotting in extracts from Ba71V-infected Vero cells and by enzyme-linked immunosorbent assay (ELISA). Prior to the first injection, sera were collected as negative controls (preimmune serum [PS]). Anti-ASFV-DP sera were tested by immunoprecipitation assays of Ba71V-infected Vero cells, and the gel band obtained was sequenced by liquid chromatography-tandem mass spectrometry (LC–MS-MS) (Ion Trap). This analysis was carried out in the Centro de Biología Molecular Severo Ochoa (CBMSO) Protein Chemistry Facility (Madrid, Spain), a regulation member of the ProteoRed network.

### Polysome profile assays.

Polysome profile analysis was carried out as previously described (30). Briefly, 10^6^ Vero cells were infected with ASFV or mock infected and harvested at the indicated times postinfection. Cell monolayers were collected, centrifuged at 1,300 × *g* for 5 min, washed with PBS supplemented with 100 μg/ml CHX, and resuspended in lysis buffer (15 mM Tris-HCl, pH 7.5, 80 mM KCl, 5 mM MgCl, 1% Triton X-100, 1% NP-40, 100 μg/ml CHX, 40 U/ml RNasin, and 1× protease inhibitor cocktail [complete Mini EDTA-free protease inhibitor cocktail tablets; Roche]). For polysome profile analysis, 10 to 50% sucrose gradients in buffer A (10 mM Tris-HCl, pH 7.4, 80 mM KCl, 10 mM MgCl_2_) supplemented with 100 μg/ml CHX were used. Samples were centrifuged for polysome separation at 270,000 × *g* for 2.5 h at 4°C in an SW40Ti rotor (Beckman Coulter). The gradients were fractionated in an Isco density gradient fractionator, and the polysome profile was obtained by continuous measurement of 254-nm absorbance in an Isco UA-5 UV monitor; data were collected with PicoLog software. For fraction collection, gradients were fractionated in a UA-6 UV-visible (Vis) detector (Teledyne Isco), which allowed the recovery of each fraction after polysome profile analysis. Study of the proteins present in the fractions was done by direct loading of 40 μl of each sample in a bis-acrylamide gel and a Western blotting procedure.

### In silico analysis.

The amino acid sequence of the Nudix motif from ASFV-DP (pD250R/Ba71V) was analyzed using Clustal-Ω software (http://www.ebi.ac.uk/Tools/msa/clustalo/) from the European Molecular Biology Laboratory (EMBL) (Heidelberg, Germany)-EBI (European Bioinformatics Institute), compared with other described eukaryotic and viral decapping enzymes: human Dcp2, SpDcp2, ScDcp2, and D9 and D10 from VACV. The three-dimensional structural prediction of ASFV-DP was generated by the Bioinformatics Service of the CBMSO (Madrid, Spain) based on the S. pombe Dcp2 crystal structure (55) and visualized using PyMol software (Schrödinger). The prediction of RNA-binding surfaces was carried out using BindUP software (65), which employs the local electrostatic potential of the protein surface to identify protein regions with potential to interact with RNA. The position of m^7^GpppG within the ASFV-DP structure was estimated by aligning the viral protein to Dcp2 in the Dcp1-Dcp2-PNRC2-cap analog complex (PDB 5KQ4) (47).

### Immunofluorescence microscopy and FISH.

FISH was performed as described in reference 5. Vero and COS-7 cell monolayers were seeded on cover glasses, washed 3 times with cold 1× PBS, and fixed with 4% paraformaldehyde. The cells were permeabilized for 15 min with 0.2% Triton X-100 in PBS and blocked with 1% bovine serum albumin (BSA) in PBS for 30 min. Anti-HA (1/500; Roche; 11867423001), anti-calnexin (1/200; StressMarq; SPC-108B), and anti-cap (1/100; Synaptic Systems; 201 001) were used as primary antibodies, in addition to two antibodies generated in our laboratory to recognize viral proteins p72 (1/500; 17LD3; Ingenasa) (66) and ASFV-DP (1/250; discussed above). Alexa Fluor-488 anti-rabbit and Alexa Fluor-555 anti-mouse (1/500; Thermo Fisher) were used as secondary antibodies as appropriate for reactivity with primary antibodies. To-Pro 3 was used to stain cellular and viral DNAs. After successive washes with distilled water and ethanol, cover glasses were fixed to microscope slides with Prolong Gold (Invitrogen) or DAPI–Fluoromount-G (SouthernBiotech) and analyzed by confocal laser scanning microscopy (CLSM) using a confocal LSM710 coupled to an AxioImager M2 microscope (Zeiss). Images were analyzed using Fiji software (67).

### Dual-fluorescence method.

The cross-linking methods used in this project are detailed below. For cCL, cell monolayers were washed twice with PBS at room temperature and then irradiated at 256 nm (0.15 J/cm^2^) on ice in the absence of PBS. Immediately after cross-linking, cold PBS was added to the cells, which were maintained at 4°C until collection (42). Cross-linking was performed with a SpectroLinker XL-1000 or XL-1500 (Spectronics Corp.). Photoactivatable ribonucleoside-enhanced cross-linking (PAR-CL) was done following described protocols (42, 43): 100 μM 4-SU was added to plates for 2 to 16 h, and protein-RNA were cross-linked after irradiation with 365 nm UV light. After PAR-CL, samples were recovered following the same protocol as for cCL. For the dual-fluorescence analysis, 1.2 × 10^7^ to 2.5 × 10^7^ stable HeLa cells were induced with 1 μg/ml Tet for 20 h before harvesting. Samples were cross-linked by conventional or PAR methods and recovered for analysis as previously described (42). Briefly, GFP fusion proteins were immunoprecipitated using GFP-Trap_A beads, and the resulting complexes were incubated with oligo(dT)-Texas Red probe. Thus, green and red signals served as proxies for protein and RNA, respectively. RNA binding was demonstrated by sensitivity of the red channel to RNases. For the analysis of RNA bound to GFP–ASFV-DP wt, GFP–ASFV-DP REGG, and GFP–ASFV-DP EEQQ, values were represented as GFP signal/Texas Red signal in order to determine the amount of RNA per amount of protein. For the analysis of GFP–ASFV-DP N-term, GFP–ASFV-DP C-term, and GFP–ASFV-DP ΔNudix, values were represented as with RNase/without RNase treatment to avoid overrepresentation of the Texas Red signal, as expression of the proteins (and consequently the GFP signal) was lower than the signal obtained for the other constructs. All experimental values are expressed as means and standard deviations (SD). Statistical analysis was performed using Student's *t* test, with the cutoff for statistical significance set at a *P* value of <0.05.

### Pulldown assays.

For pulldown assays, GST or GST–ASFV-DP fusion proteins were expressed in E. coli BL21 as described above and purified with glutathione Sepharose beads (GE Healthcare). Both proteins were incubated with extracts of mock- or ASFV-infected Vero cells at 6 or 18 hpi, and after intensive washing, peptides associated with the GST complexes were sequenced by mass spectrometry (at the Proteomics Core Facility of CBMSO, Madrid, Spain). Briefly, equal quantities of the protein extracts were suspended in a volume of up to 50 μl of sample buffer and then applied to 1.2-cm-wide wells of a conventional SDS-PAGE gel (0.75 mm thick; 4% stacking and 10% resolving). The run was stopped as soon as the front entered 3 mm into the resolving gel, so that the whole proteome became concentrated in the stacking/resolving gel interface. The unseparated protein bands were visualized by Coomassie staining, excised, cut into cubes (2 by 2 mm), and placed in 0.5-ml microcentrifuge tubes (68). The gel pieces were destained in acetonitrile (ACN)-water (1:1), reduced and alkylated (disulfide bonds from cysteinyl residues were reduced with 10 mM dithiothreitol [DTT] for 1 h at 56°C, and then the thiol groups were alkylated with 50 mM iodoacetamide for 1 h at room temperature in darkness), and digested *in situ* with sequencing-grade trypsin (Promega, Madison, WI) as described by Shevchenko et al. (69) with minor modifications. The gel pieces were shrunk by removing all liquid using sufficient ACN. The acetonitrile was pipetted out, and the gel pieces were dried in a SpeedVac. The dried gel pieces were reswollen in 50 mM ammonium bicarbonate, pH 8.8, with 60 ng/μl trypsin at a 5:1 (wt/wt) protein/trypsin ratio. The tubes were kept on ice for 2 h and incubated at 37°C for 12 h. Digestion was stopped by the addition of 1% trifluoroacetic acid (TFA). Whole supernatants were dried down and then desalted onto ZipTip C_18_ pipette tips (Millipore) until the mass spectrometric analyses, which were performed for the three samples consecutively. The desalted protein digest was dried, resuspended in 8 μl of 0.1% formic acid, and analyzed by reverse phase (RP)–LC–MS-MS in an Agilent 1100 system coupled to a linear ion trap LTQ-Velos mass spectrometer (Thermo Scientific, Waltham, MA, USA). The peptides were separated by reverse phase chromatography using a 0.18-mm by 150-mm Bio-Basic C_18_ RP column (Thermo Scientific) operating at 1.8 μl/min. Peptides were eluted using a 35-min gradient from 5 to 40% solvent B (solvent A, 0,1% formic acid in water; solvent B, 0,1% formic acid, 80% acetonitrile in water). Electrospray ionization (ESI) was done using a microspray metal needle kit (Thermo Scientific) interface. Peptides were detected in survey scans from 400 to 1,600 atomic mass units (amu) (1 microscan), followed by 10 data-dependent MS-MS scans (Top 10), using an isolation width of 2 u (in mass/charge ratio units), a normalized collision energy of 35%, and dynamic exclusion applied in 30-s periods. Peptide identification from raw data was carried out using the SEQUEST algorithm (Proteome Discoverer 1.4; Thermo Scientific). A database search was performed against UniProt_VPPA_Ba71v, UniProt_Primates, and local databases. The following constraints were used for the searches: tryptic cleavage after Arg and Lys, up to two missed cleavage sites, and tolerances of 1 Da for precursor ions and 0.8 Da for MS-MS fragment ions. The searches were performed allowing optional Met oxidation and Cys carbamidomethylation. Searches against a decoy database (the integrated decoy approach) used a false-discovery rate (FDR) of <0.01. Enriched protein analyses after pulldown assays were done on the ConsensusPath.db website (Bioinformatics Group of the Vertebrate Genomics Department at the Max-Planck-Institute for Molecular Genetics in Berlin, Germany [http://cpdb.molgen.mpg.de/]).

### Immunoprecipitation assays.

For immunoprecipitation assays, protein A/G agarose beads (Pierce) were washed twice with PBS, equilibrated with modified radioimmunoprecipitation assay (RIPA) buffer (mRIPA) (50 mM Tris-HCl, pH 7.5, 1% NP-40, 0.25% sodium deoxycholate, 150 mM NaCl, 1 mM EDTA, 1× protease inhibitors [Roche], and 1× phosphatase inhibitors [PhosStop Easy pack; Roche]), and incubated for 1 h at 4°C with specific antisera. Next, extracts from 10^7^ mock- or Ba71V-infected (MOI = 5 PFU/cell) Vero cells were lysed with mRIPA and incubated with the beads for 2 h at 4°C with rotation. The beads were intensively washed with 0.05% PBS-Tween, and proteins were eluted with Laemmli buffer (10 mM Tris-HCl, pH 6.8, 10% glycerol, 2% SDS, 0.1% 2-mercaptoethanol, and 0.0005% bromophenol blue) at 70°C for 5 min and finally analyzed by Western blotting as described below. RNase treatment was carried out by incubating samples prior to IP with 350 U of RNase T1 and 140 mg/ml of RNase A for 15 min at room temperature.

For immunoprecipitation and RT-qPCR assays, we optimized the GFP Trap_A bead immunoprecipitation protocol (70). After immunoprecipitation, RNA was extracted from protein complexes and analyzed by RT-qPCR. Briefly, 1.2 × 10^7^ HeLa eGFP, 2.4 × 10^7^ HeLa MOV10-YFP, and 4.8 × 10^7^ HeLa GFP–ASFV-DP wt cells were treated with Tet (0.6 μg/ml, 1.2 μg/ml, and 1.2 μg/ml, respectively) for 20 h to induce protein expression and then conventionally cross-linked and harvested as previously described. After the cells were resuspended in 0.3 to 1.2 ml of lysis buffer (100 mM KCl, 5 mM MgCl_2_, 10 mM Tris, pH 7.5, 0.5% NP-40, 1 mM DTT, 100 units/ml RNasin, 1× phenylmethylsulfonyl fluoride [PMSF], and 200 μM ribonucleoside vanadyl complex) and mixed with dilution buffer (500 mM NaCl, 1 mM MgCl_2_, 0.05% SDS, 0.025% NP-40, 50 mM Tris-HCl, pH 7.5, 1/500 RNasin, 1× PMSF, and 200 μM ribonucleoside vanadyl complex), 10% of the sample was removed as input and stored at 4°C until RNA extraction. Samples were incubated with 20 μl of GFP-Trap_A beads for 4 h at 4°C with rotation and washed as follows: twice (2 min each time) with wash buffer 1 (500 mM NaCl, 1 mM MgCl_2_, 20 mM Tris-HCl, pH 7.5, 0.025% NP-40, 1/500 RNasin, and PMSF), twice (2 min each time) with wash buffer 2 (250 mM NaCl, 1 mM MgCl_2_, 20 mM Tris-HCl, pH 7.5, 0.025% NP-40, 1/500 RNasin, and PMSF), and twice (2 min each time) with wash buffer 3 (150 mM NaCl, 1 mM MgCl_2_, 20 mM Tris-HCl, pH 7.5, 0.025% NP-40, 1/500 RNasin, and PMSF). After the final wash, 3 μl was tested by Western blotting. To elute the immunoprecipitated proteins, beads were mixed with 2× Laemmli buffer and boiled for 5 min at 95°C; GFP complexes were detected using a specific anti-GFP antibody. Elution of RNA from protein complexes was done by direct treatment of the beads with proteinase K; the beads were incubated with 1× proteinase K buffer (50 mM Tris HCl, pH 7.5, 750 mM NaCl, 1% SDS, 50 mM EDTA, 2.5 mM DTT, and 25 mM CaCl_2_) and 10 μg proteinase K in a final volume of 100 μl for 1 h at 37°C with strong shaking. RNA was extracted with phenol-chloroform-isoamyl alcohol and analyzed by qPCR as described below. Immunoprecipitated eGFP was used as a calibrator, and samples were normalized to total RNA (input).

### qPCR.

The isolated RNA was retrotranscribed to cDNA using retrotranscriptase enzyme (Roche) following the manufacturer′s instructions. cDNA was generated from 1 μg of RNA from cell lysates and all available RNA from immunoprecipitation samples, and qPCR analysis was performed with a MicroAmp Optical 384-well reaction plate with barcode and Fast Sybr Green master mix (Life Technologies). To quantify expression of genes from cell extracts, we used 5 ng cDNA per reaction as the template (in triplicate); to quantify and analyze RNA bound to ASFV-DPs, we amplified all cDNA obtained after the IP. For each gene, 1× Sybr Green master mix and 0.25 mM forward and reverse primers (18S FW, 18S RV, β-actin FW, β-actin RV, BIRC2 FW, BIRC2 RV, eIF4A1 FW, eIF4A1 RV, eIF4E FW, eIF4E RV, p53 FW, p53 RV, A238L FW, A238L RV, A224L FW, A224L RV, B646L FW, and B646L RV [Table 3]) were used. The plates were covered with MicroAmp Optical adhesive films (Life Technologies), and qPCRs were run as follows: 1 cycle of 20 s at 95°C and 40 cycles at two temperatures (10 s at 95°C and 20 s at 60°C) in an ABI Prism 7900HT SDS thermocycler (Applied Biosystems). In the case of RNA extracted from cells, values were normalized to the 18S gene, while values of RNA extracted from immunoprecipitated proteins were normalized to the starting quantity of RNA (input RNA). Experimental values are expressed as means and SD or standard error of the mean (SEM), as indicated. Statistical analysis was performed by Student′s *t* test, and the cutoff for statistical significance was set at a *P* value of <0.05.

### Western blot analysis.

Total protein extracts were obtained from mock- or ASFV-infected cells and lysed in mRIPA. Quantification of total protein was done using a bicinchoninic acid (BCA) protein assay kit (Pierce) following the manufacturer′s protocol. For protein electrophoretic separation assays, 10 to 60 μg total protein was mixed with Laemmli buffer and electrophoresed in SDS-PAGE gels under reducing conditions. Following separation, proteins were transferred to Immobilon-P membranes (Millipore) and incubated with primary antibodies: anti-ASFV-DP or anti-p72 (1/1,000; generated in our laboratory), anti-actin (1/1,000; Santa Cruz Biotechnology), anti-tubulin (1/250; Sigma-Aldrich), anti-GFP (1/1,000; Roche), anti-PABP (1/200; Abcam), anti-RPS6 (1/1,000; 5G10; Cell Signaling), anti-RPL24 and anti-RPS6 (1/1,000; a kind gift from Anne Willis, MRC Toxicology Unit, Leicester, United Kingdom), or anti-RPL23a (1/500; a kind gift from Miguel Angel Rodriguez Gabriel, CBMSO, Madrid, Spain [71]), and an antibody able to recognize the inducible proteins of ASFV or anti-ASFV proteins (1/4,000; generated at CBMSO [72]). For detection, the chemiluminescent ECL Western blotting analysis system (Amersham Pharmacia) was used, following the manufacturer′s instructions.

## Supplementary Material

Supplemental material

## References

[1] Waterhouse PM, Wang MB, Lough T 2001 Gene silencing as an adaptive defence against viruses. Nature 411:834–842. doi:10.1038/35081168. 11459066

[2] Pichlmair A, Schulz O, Tan CP, Naslund TI, Liljestrom P, Weber F, Reis e Sousa C 2006 RIG-I-mediated antiviral responses to single-stranded RNA bearing 5′-phosphates. Science 314:997–1001. doi:10.1126/science.1132998. 17038589

[3] Rigby RE, Rehwinkel J 2015 RNA degradation in antiviral immunity and autoimmunity. Trends Immunol 36:179–188. doi:10.1016/j.it.2015.02.001. 25709093 PMC4358841

[4] Abernathy E, Glaunsinger B 2015 Emerging roles for RNA degradation in viral replication and antiviral defense. Virology 479-480: 600–608. doi:10.1016/j.virol.2015.02.007. 25721579 PMC4424162

[5] Sanchez EG, Quintas A, Nogal M, Castello A, Revilla Y 2013 African swine fever virus controls the host transcription and cellular machinery of protein synthesis. Virus Res 173:58–75. doi:10.1016/j.virusres.2012.10.025. 23154157

[6] Reineke LC, Lloyd RE 2013 Diversion of stress granules and P-bodies during viral infection. Virology 436:255–267. doi:10.1016/j.virol.2012.11.017. 23290869 PMC3611887

[7] Sandri-Goldin RM 2011 The many roles of the highly interactive HSV protein ICP27, a key regulator of infection. Future Microbiol 6:1261–1277. doi:10.2217/fmb.11.119. 22082288

[8] Blanchette P, Kindsmuller K, Groitl P, Dallaire F, Speiseder T, Branton PE, Dobner T 2008 Control of mRNA export by adenovirus E4orf6 and E1B55K proteins during productive infection requires E4orf6 ubiquitin ligase activity. J Virol 82:2642–2651. doi:10.1128/JVI.02309-07. 18184699 PMC2258987

[9] von Kobbe C, van Deursen JM, Rodrigues JP, Sitterlin D, Bachi A, Wu X, Wilm M, Carmo-Fonseca M, Izaurralde E 2000 Vesicular stomatitis virus matrix protein inhibits host cell gene expression by targeting the nucleoporin Nup98. Mol Cell 6:1243–1252. doi:10.1016/S1097-2765(00)00120-9. 11106761

[10] Park N, Katikaneni P, Skern T, Gustin KE 2008 Differential targeting of nuclear pore complex proteins in poliovirus-infected cells. J Virol 82:1647–1655. doi:10.1128/JVI.01670-07. 18045934 PMC2258732

[11] Castello A, Izquierdo JM, Welnowska E, Carrasco L 2009 RNA nuclear export is blocked by poliovirus 2A protease and is concomitant with nucleoporin cleavage. J Cell Sci 122:3799–3809. doi:10.1242/jcs.055988. 19789179

[12] Satterly N, Tsai PL, van Deursen J, Nussenzveig DR, Wang Y, Faria PA, Levay A, Levy DE, Fontoura BM 2007 Influenza virus targets the mRNA export machinery and the nuclear pore complex. Proc Natl Acad Sci U S A 104:1853–1858. doi:10.1073/pnas.0610977104. 17267598 PMC1794296

[13] Feng P, Everly DN Jr, Read GS 2005 mRNA decay during herpes simplex virus (HSV) infections: protein-protein interactions involving the HSV virion host shutoff protein and translation factors eIF4H and eIF4A. J Virol 79:9651–9664. doi:10.1128/JVI.79.15.9651-9664.2005. 16014927 PMC1181552

[14] Covarrubias S, Gaglia MM, Kumar GR, Wong W, Jackson AO, Glaunsinger BA 2011 Coordinated destruction of cellular messages in translation complexes by the gammaherpesvirus host shutoff factor and the mammalian exonuclease Xrn1. PLoS Pathog 7:e1002339. doi:10.1371/journal.ppat.1002339. 22046136 PMC3203186

[15] Richner JM, Clyde K, Pezda AC, Cheng BY, Wang T, Kumar GR, Covarrubias S, Coscoy L, Glaunsinger B 2011 Global mRNA degradation during lytic gammaherpesvirus infection contributes to establishment of viral latency. PLoS Pathog 7:e1002150. doi:10.1371/journal.ppat.1002150. 21811408 PMC3141057

[16] Huang C, Lokugamage KG, Rozovics JM, Narayanan K, Semler BL, Makino S 2011 SARS coronavirus nsp1 protein induces template-dependent endonucleolytic cleavage of mRNAs: viral mRNAs are resistant to nsp1-induced RNA cleavage. PLoS Pathog 7:e1002433. doi:10.1371/journal.ppat.1002433. 22174690 PMC3234236

[17] Jagger BW, Wise HM, Kash JC, Walters KA, Wills NM, Xiao YL, Dunfee RL, Schwartzman LM, Ozinsky A, Bell GL, Dalton RM, Lo A, Efstathiou S, Atkins JF, Firth AE, Taubenberger JK, Digard P 2012 An overlapping protein-coding region in influenza A virus segment 3 modulates the host response. Science 337:199–204. doi:10.1126/science.1222213. 22745253 PMC3552242

[18] McLennan AG 2007 Decapitation: poxvirus makes RNA lose its head. Trends Biochem Sci 32:297–299. doi:10.1016/j.tibs.2007.05.001. 17498957

[19] Parrish S, Moss B 2007 Characterization of a second vaccinia virus mRNA-decapping enzyme conserved in poxviruses. J Virol 81:12973–12978. doi:10.1128/JVI.01668-07. 17881455 PMC2169080

[20] Parrish S, Resch W, Moss B 2007 Vaccinia virus D10 protein has mRNA decapping activity, providing a mechanism for control of host and viral gene expression. Proc Natl Acad Sci U S A 104:2139–2144. doi:10.1073/pnas.0611685104. 17283339 PMC1793903

[21] Vinuela E 1985 African swine fever virus. Curr Top Microbiol Immunol 116:151–170. 3893908 10.1007/978-3-642-70280-8_8

[22] Costard S, Mur L, Lubroth J, Sanchez-Vizcaino JM, Pfeiffer DU 2013 Epidemiology of African swine fever virus. Virus Res 173:191–197. doi:10.1016/j.virusres.2012.10.030. 23123296

[23] Heath CM, Windsor M, Wileman T 2001 Aggresomes resemble sites specialized for virus assembly. J Cell Biol 153:449–455. doi:10.1083/jcb.153.3.449. 11331297 PMC2190574

[24] Rodriguez JM, Salas ML 2013 African swine fever virus transcription. Virus Res 173:15–28. doi:10.1016/j.virusres.2012.09.014. 23041356

[25] Rodriguez JM, Salas ML, Santaren JF 2001 African swine fever virus-induced polypeptides in porcine alveolar macrophages and in Vero cells: two-dimensional gel analysis. Proteomics 1:1447–1456. doi:10.1002/1615-9861(200111)1:11<1447::AID-PROT1447>3.0.CO;2-Y. 11922604

[26] Castello A, Quintas A, Sanchez EG, Sabina P, Nogal M, Carrasco L, Revilla Y 2009 Regulation of host translational machinery by African swine fever virus. PLoS Pathog 5:e1000562. doi:10.1371/journal.ppat.1000562. 19714237 PMC2727446

[27] Granja AG, Nogal ML, Hurtado C, Salas J, Salas ML, Carrascosa AL, Revilla Y 2004 Modulation of p53 cellular function and cell death by African swine fever virus. J Virol 78:7165–7174. doi:10.1128/JVI.78.13.7165-7174.2004. 15194793 PMC421689

[28] Parrish S, Hurchalla M, Liu SW, Moss B 2009 The African swine fever virus g5R protein possesses mRNA decapping activity. Virology 393:177–182. doi:10.1016/j.virol.2009.07.026. 19695654 PMC3392020

[29] Cartwright JL, Safrany ST, Dixon LK, Darzynkiewicz E, Stepinski J, Burke R, McLennan AG 2002 The g5R (D250) gene of African swine fever virus encodes a Nudix hydrolase that preferentially degrades diphosphoinositol polyphosphates. J Virol 76:1415–1421. doi:10.1128/JVI.76.3.1415-1421.2002. 11773415 PMC135849

[30] Martinez-Azorin F, Remacha M, Ballesta JP 2008 Functional characterization of ribosomal P1/P2 proteins in human cells. Biochem J 413:527–534. doi:10.1042/BJ20080049. 18422483

[31] Schneider-Poetsch T, Ju J, Eyler DE, Dang Y, Bhat S, Merrick WC, Green R, Shen B, Liu JO 2010 Inhibition of eukaryotic translation elongation by cycloheximide and lactimidomycin. Nat Chem Biol 6:209–217. doi:10.1038/nchembio.304. 20118940 PMC2831214

[32] Reid DW, Nicchitta CV 2015 Diversity and selectivity in mRNA translation on the endoplasmic reticulum. Nat Rev Mol Cell Biol 16:221–231. doi:10.1038/nrm3958. 25735911 PMC4494666

[33] Salas ML, Andres G 2013 African swine fever virus morphogenesis. Virus Res 173:29–41. doi:10.1016/j.virusres.2012.09.016. 23059353

[34] Epifano C, Krijnse-Locker J, Salas ML, Rodriguez JM, Salas J 2006 The African swine fever virus nonstructural protein pB602L is required for formation of the icosahedral capsid of the virus particle. J Virol 80:12260–12270. doi:10.1128/JVI.01323-06. 17035321 PMC1676282

[35] Garcia-Escudero R, Andres G, Almazan F, Vinuela E 1998 Inducible gene expression from African swine fever virus recombinants: analysis of the major capsid protein p72. J Virol 72:3185–3195. 9580160 10.1128/jvi.72.4.3185-3195.1998 PMC109780

[36] Bochnig P, Reuter R, Bringmann P, Luhrmann R 1987 A monoclonal antibody against 2,2,7-trimethylguanosine that reacts with intact, class U, small nuclear ribonucleoproteins as well as with 7-methylguanosine-capped RNAs. Eur J Biochem 168:461–467. doi:10.1111/j.1432-1033.1987.tb13439.x. 2959477

[37] Vogt C, Preuss E, Mayer D, Weber F, Schwemmle M, Kochs G 2008 The interferon antagonist ML protein of thogoto virus targets general transcription factor IIB. J Virol 82:11446–11453. doi:10.1128/JVI.01284-08. 18768974 PMC2573285

[38] Maldonado E, Cabrejos ME, Banks L, Allende JE 2002 Human papillomavirus-16 E7 protein inhibits the DNA interaction of the TATA binding transcription factor. J Cell Biochem 85:663–669. doi:10.1002/jcb.10172. 11968006

[39] Kalveram B, Lihoradova O, Ikegami T 2011 NSs protein of Rift Valley fever virus promotes posttranslational downregulation of the TFIIH subunit p62. J Virol 85:6234–6243. doi:10.1128/JVI.02255-10. 21543505 PMC3126510

[40] Kundu P, Raychaudhuri S, Tsai W, Dasgupta A 2005 Shutoff of RNA polymerase II transcription by poliovirus involves 3C protease-mediated cleavage of the TATA-binding protein at an alternative site: incomplete shutoff of transcription interferes with efficient viral replication. J Virol 79:9702–9713. doi:10.1128/JVI.79.15.9702-9713.2005. 16014932 PMC1181600

[41] Dasgupta A, Scovell WM 2003 TFIIA abrogates the effects of inhibition by HMGB1 but not E1A during the early stages of assembly of the transcriptional preinitiation complex. Biochim Biophys Acta 1627:101–110. doi:10.1016/S0167-4781(03)00080-0. 12818428

[42] Strein C, Alleaume AM, Rothbauer U, Hentze MW, Castello A 2014 A versatile assay for RNA-binding proteins in living cells. RNA 20:721–731. doi:10.1261/rna.043562.113. 24664470 PMC3988573

[43] Castello A, Fischer B, Eichelbaum K, Horos R, Beckmann BM, Strein C, Davey NE, Humphreys DT, Preiss T, Steinmetz LM, Krijgsveld J, Hentze MW 2012 Insights into RNA biology from an atlas of mammalian mRNA-binding proteins. Cell 149:1393–1406. doi:10.1016/j.cell.2012.04.031. 22658674

[44] Matia-Gonzalez AM, Iadevaia V, Gerber AP 2017 A versatile tandem RNA isolation procedure to capture in vivo formed mRNA-protein complexes. Methods 118-119: 93–100. doi:10.1016/j.ymeth.2016.10.005. 27746303

[45] Dunckley T, Parker R 1999 The DCP2 protein is required for mRNA decapping in Saccharomyces cerevisiae and contains a functional MutT motif. EMBO J 18:5411–5422. doi:10.1093/emboj/18.19.5411. 10508173 PMC1171610

[46] Song MG, Bail S, Kiledjian M 2013 Multiple Nudix family proteins possess mRNA decapping activity. RNA 19:390–399. doi:10.1261/rna.037309.112. 23353937 PMC3677249

[47] Mugridge JS, Ziemniak M, Jemielity J, Gross JD 2016 Structural basis of mRNA-cap recognition by Dcp1-Dcp2. Nat Struct Mol Biol 23:987–994. doi:10.1038/nsmb.3301. 27694842 PMC5113729

[48] Deshmukh MV, Jones BN, Quang-Dang DU, Flinders J, Floor SN, Kim C, Jemielity J, Kalek M, Darzynkiewicz E, Gross JD 2008 mRNA decapping is promoted by an RNA-binding channel in Dcp2. Mol Cell 29:324–336. doi:10.1016/j.molcel.2007.11.027. 18280238

[49] She M, Decker CJ, Svergun DI, Round A, Chen N, Muhlrad D, Parker R, Song H 2008 Structural basis of dcp2 recognition and activation by dcp1. Mol Cell 29:337–349. doi:10.1016/j.molcel.2008.01.002. 18280239 PMC2323275

[50] Schneider RJ, Mohr I 2003 Translation initiation and viral tricks. Trends Biochem Sci 28:130–136. doi:10.1016/S0968-0004(03)00029-X. 12633992

[51] Narayanan K, Makino S 2013 Interplay between viruses and host mRNA degradation. Biochim Biophys Acta 1829:732–741. doi:10.1016/j.bbagrm.2012.12.003. 23274304 PMC3632658

[52] Parrish S, Moss B 2006 Characterization of a vaccinia virus mutant with a deletion of the D10R gene encoding a putative negative regulator of gene expression. J Virol 80:553–561. doi:10.1128/JVI.80.2.553-561.2006. 16378957 PMC1346865

[53] Katsafanas GC, Moss B 2007 Colocalization of transcription and translation within cytoplasmic poxvirus factories coordinates viral expression and subjugates host functions. Cell Host Microbe 2:221–228. doi:10.1016/j.chom.2007.08.005. 18005740 PMC2084088

[54] Song MG, Li Y, Kiledjian M 2010 Multiple mRNA decapping enzymes in mammalian cells. Mol Cell 40:423–432. doi:10.1016/j.molcel.2010.10.010. 21070968 PMC2982215

[55] She M, Decker CJ, Chen N, Tumati S, Parker R, Song H 2006 Crystal structure and functional analysis of Dcp2p from Schizosaccharomyces pombe. Nat Struct Mol Biol 13:63–70. doi:10.1038/nsmb1033. 16341225 PMC1952686

[56] Piccirillo C, Khanna R, Kiledjian M 2003 Functional characterization of the mammalian mRNA decapping enzyme hDcp2. RNA 9:1138–1147. doi:10.1261/rna.5690503. 12923261 PMC1370477

[57] Souliere MF, Perreault JP, Bisaillon M 2009 Characterization of the vaccinia virus D10 decapping enzyme provides evidence for a two-metal-ion mechanism. Biochem J 420:27–35. doi:10.1042/BJ20082296. 19210265

[58] Liu SW, Katsafanas GC, Liu R, Wyatt LS, Moss B 2015 Poxvirus decapping enzymes enhance virulence by preventing the accumulation of dsRNA and the induction of innate antiviral responses. Cell Host Microbe 17:320–331. doi:10.1016/j.chom.2015.02.002. 25766293 PMC4359750

[59] Esclatine A, Taddeo B, Roizman B 2004 The UL41 protein of herpes simplex virus mediates selective stabilization or degradation of cellular mRNAs. Proc Natl Acad Sci U S A 101:18165–18170. doi:10.1073/pnas.0408272102. 15596716 PMC539803

[60] Guerra S, Lopez-Fernandez LA, Pascual-Montano A, Munoz M, Harshman K, Esteban M 2003 Cellular gene expression survey of vaccinia virus infection of human HeLa cells. J Virol 77:6493–6506. doi:10.1128/JVI.77.11.6493-6506.2003. 12743306 PMC154985

[61] Li Y, Song MG, Kiledjian M 2008 Transcript-specific decapping and regulated stability by the human Dcp2 decapping protein. Mol Cell Biol 28:939–948. doi:10.1128/MCB.01727-07. 18039849 PMC2223397

[62] Li Y, Ho ES, Gunderson SI, Kiledjian M 2009 Mutational analysis of a Dcp2-binding element reveals general enhancement of decapping by 5′-end stem-loop structures. Nucleic Acids Res 37:2227–2237. doi:10.1093/nar/gkp087. 19233875 PMC2673433

[63] Dulbecco R, Freeman G 1959 Plaque production by the polyoma virus. Virology 8:396–397. doi:10.1016/0042-6822(59)90043-1. 13669362

[64] Enjuanes L, Carrascosa AL, Moreno MA, Vinuela E 1976 Titration of African swine fever (ASF) virus. J Gen Virol 32:471–477. doi:10.1099/0022-1317-32-3-471. 823294

[65] Paz I, Kligun E, Bengad B, Mandel-Gutfreund Y 2016 BindUP: a web server for non-homology-based prediction of DNA and RNA binding proteins. Nucleic Acids Res 44:W568–W574. doi:10.1093/nar/gkw454. 27198220 PMC4987955

[66] Sanz A, Garcia-Barreno B, Nogal ML, Vinuela E, Enjuanes L 1985 Monoclonal antibodies specific for African swine fever virus proteins. J Virol 54:199–206. 3882998 10.1128/jvi.54.1.199-206.1985 PMC254778

[67] Schindelin J, Arganda-Carreras I, Frise E, Kaynig V, Longair M, Pietzsch T, Preibisch S, Rueden C, Saalfeld S, Schmid B, Tinevez JY, White DJ, Hartenstein V, Eliceiri K, Tomancak P, Cardona A 2012 Fiji: an open-source platform for biological-image analysis. Nat Methods 9:676–682. doi:10.1038/nmeth.2019. 22743772 PMC3855844

[68] Moreno ML, Escobar J, Izquierdo-Alvarez A, Gil A, Perez S, Pereda J, Zapico I, Vento M, Sabater L, Marina A, Martinez-Ruiz A, Sastre J 2014 Disulfide stress: a novel type of oxidative stress in acute pancreatitis. Free Radic Biol Med 70:265–277. doi:10.1016/j.freeradbiomed.2014.01.009. 24456905

[69] Shevchenko A, Wilm M, Vorm O, Mann M 1996 Mass spectrometric sequencing of proteins silver-stained polyacrylamide gels. Anal Chem 68:850–858. doi:10.1021/ac950914h. 8779443

[70] Rothbauer U, Zolghadr K, Muyldermans S, Schepers A, Cardoso MC, Leonhardt H 2008 A versatile nanotrap for biochemical and functional studies with fluorescent fusion proteins. Mol Cell Proteomics 7:282–289. doi:10.1074/mcp.M700342-MCP200. 17951627

[71] Guarinos E, Santos C, Sanchez A, Qiu DY, Remacha M, Ballesta JP 2003 Tag-mediated fractionation of yeast ribosome populations proves the monomeric organization of the eukaryotic ribosomal stalk structure. Mol Microbiol 50:703–712. doi:10.1046/j.1365-2958.2003.03733.x. 14617190

[72] del Val M, Vinuela E 1987 Glycosylated components induced in African swine fever (ASF) virus-infected Vero cells. Virus Res 7:297–308. doi:10.1016/0168-1702(87)90044-X. 3617927

