# Supplementary material for: Characterization of the African Swine Fever Virus Decapping Enzyme during Infection
Source: J Virol. 2017 Nov 30;91(24):e00990-17. doi: 10.1128/JVI.00990-17 (PMC5709586; doi:10.1128/JVI.00990-17)
Supplement: Supplemental material [file supp_91_24_e00990-17__index.html]

Supplemental material 

# Characterization of the African Swine Fever Virus Decapping Enzyme during Infection

## Supplemental material

- Supplemental file 1 -

  Fig. S1 (ASFV decapping protein interacts with cellular translation machinery.)

  Fig. S2 (Effect of 4-thiouridine on ASFV infection.)

  Table S1 (Identification of cellular proteins interacting with ASFV decapping protein.)

  Table S2 (Enriched pathway, pathway members, and *q* value for each sample).

  PDF, 10M
