# Supplementary material for: Characterization of the African Swine Fever Virus Decapping Enzyme during Infection
Source: J Virol. 2017 Nov 30;91(24):e00990-17. doi: 10.1128/JVI.00990-17 (PMC5709586; doi:10.1128/JVI.00990-17)
Supplement: Supplemental material [file JVI.00990-17_zjv024173173s1.pdf]

Supplementary Figure 1

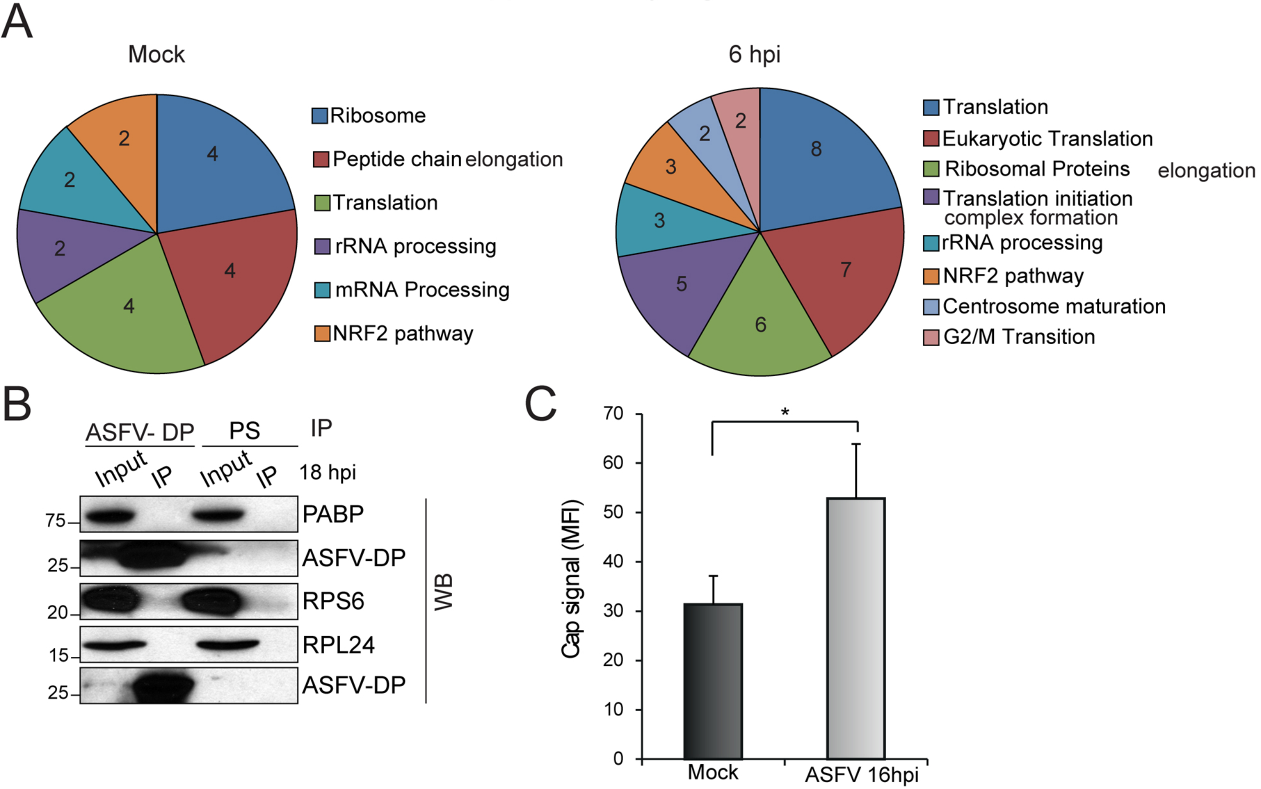

Supplementary Figure 2

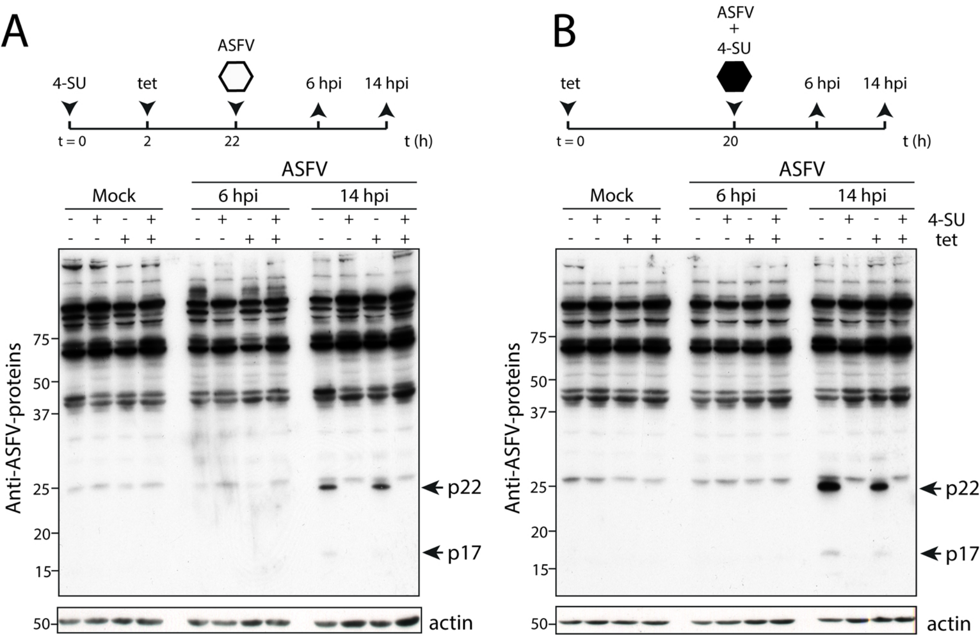

Supplementary Figure 1. ASFV-DP interacts with the cellular translation machinery A) Analysis of the proteins enriched in the GST-ASFV-DP fraction after incubation with mock- or ASFV-infected samples from 6 hpi. Numbers inside segments represent the quantity of proteins found in each category. **B)** Analysis of ASFV-DP interaction with different ribosomal proteins. Vero cells were ASFV infected (M.O.I. = 5 pfu/cell) and harvested at 18 hpi. Samples were incubated with anti-ASFV-DP or PS (preimmune serum) and PABP, ASFV-DP, RPS6 and RPL24 presence were analyzed by western blot. **C)** Quantification of the mRNA cap signal in mock- and ASFV-infected cells by ImageJ software (mean fluorescence intensity, MFI +/- S.D., n = 2). *: $p < 0.05$. Data are representative images from two separate experiments.

Supplementary Figure 2. Effect of 4-SU on ASFV infection. In order to test the side effects of 4-SU presence on ASFV infection, two different experimental procedures were carried out. In the first, **(A)** the HeLa GFP-ASFV-DP wt stable cell line was treated with 4-SU 2 h prior to induction of recombinant GFP-ASFV-DP wt protein with tetracycline (tet), and samples were recovered at the indicated times post-infection; a schematic representation of the experimental procedure is shown in the upper panel. ASFV protein levels were detected by western blot using a specific serum able to recognize viral proteins induced by the infection (anti-ASFV-proteins) (bottom panel). In the second approach, **(B)** 4-SU was added to HeLa GFP-ASFV-DP wt expressing cells together with the viral inoculum (18 h after induction of expression with tet). A schematic representation of the experimental procedure is shown in the upper panel. ASFV protein levels were determined by western blot using the anti-ASFV-proteins serum (bottom panel); actin was used as loading control.

Supplementary table 1. Identification of cellular proteins interacting with ASFV-DP. The table shows the UniProt accession number (accession), the identified protein (description), the score (score), the coverage of identification for each protein (coverage), the number of peptides identified (peptides), the number of unique peptides (unique peptides) and the peptide sequences which allowed identification of the protein (identified sequence) .

Mock

Accession	Description	Score	Coverage	Unique Peptides	Peptides	Identified sequence
F7HSL0	Carbonyl reductase [NADPH] 1 OS=Callithrix jacchus GN=CBR1 PE=2 SV=1 - [F7HSL0_CALJA]	82.14	31.77	2	7	GQAAVQQLQAEGLSPR<br>SETITEEELVGLMNK<br>FRSETITEEELVGLMNK<br>EYGGLDVLVNNAGIAFK<br>VADPTPFHIQAEVTMK<br>ILLNAccPGWVR<br>TDmAGPNATK<br>TDMAGPNATK<br>ILLNACcPGWVR<br>FRSETITEEELVGLmNK
G7NBW9	Glutathione S-transferase P (Fragment) OS=Macaca mulatta GN=EGK_05927 PE=3 SV=1 - [G7NBW9_MACMU]	76.63	40.00	3	7	FQDGDLTLYQSNTFLR<br>EAALVDmVNDGVEDLR<br>EAALVDMVNDGVEDLR<br>YLSLIYTNYEAGKDDYVK<br>PFETLLSQNQGGK<br>MLLADQGQSWK<br>YLSLIYTNYEAGK<br>AScLYGQLPK
Q5T6W2	Heterogeneous nuclear ribonucleoprotein K (Fragment) OS=Homo sapiens GN=HNRNPK PE=1 SV=1 - [Q5T6W2_	13.66	16.62	5	5	GSYGDLGGPIITTQVTIPK<br>TDYNASVSVPDSSGPER<br>GSDFDcELR<br>NTDEMVELR<br>GGDLMAYDR
B3KML9	cDNA FLJ11352 fis, clone HEMBA1000020, highly similar to Tubulin beta-2C chain OS=Homo sapiens PE=2 SV=1<br>[B3KML9_HUMAN]	12.68	10.83	1	4	ImNTFSVVPSPK<br>IMNTFSVVPSPK<br>TAVcDIPPR<br>ISEQFTAMFR<br>INVYYNEATGGK
H0YNP5	Annexin (Fragment) OS=Homo sapiens GN=ANXA2 PE=1 SV=1 - [H0YNP5_HUMAN]	9.59	21.71	3	3	AEDGSVIDYELIDQDAR<br>TNQELQEINR<br>TPAQYDASELK
Q6PKI6	YBX1 protein (Fragment) OS=Homo sapiens GN=YBX1 PE=2 SV=1 - [Q6PKI6_HUMAN]	8.13	13.53	1	2	GAEAANVTGPGGVPVQGSK<br>SVGDGETVEFDVVEGEK
H9FMA8	DNA-binding protein A isoform b (Fragment) OS=Macaca mulatta GN=CSDA PE=2 SV=1 - [H9FMA8_MACMU]	7.69	45.57	1	2	SVGDGETVEFDVVEGEK<br>GAEAANVTGPDGVPVEGSR
P62263	40S ribosomal protein S14 OS=Homo sapiens GN=RPS14 PE=1 SV=3 - [RS14_HUMAN]	7.29	22.52	3	3	TPGPGAQSALR<br>ELGITALHIK<br>IEDVTPIPSDSTR
E9PKZ0	60S ribosomal protein L8 (Fragment) OS=Homo sapiens GN=RPL8 PE=1 SV=1 - [E9PKZ0_HUMAN]	6.77	13.17	2	2	ASGNYATVISHNPETK<br>AVVGVVAGGGR
B4DEF7	cDNA FLJ60062, highly similar to 78 kDa glucose-regulated protein OS=Homo sapiens PE=2 SV=1 - [B4DEF7_HU	6.68	10.43	2	2	NQLTSNPENTVFDAK<br>ITPSYVAFTPEGER
C9J4Z3	60S ribosomal protein L37a OS=Homo sapiens GN=RPL37A PE=4 SV=1 - [C9J4Z3_HUMAN]	5.65	44.12	2	2	TVAGGAWTYNTTSAVTVK<br>AVGIWHcGScMK
Q6DCA8	BCLAF1 protein (Fragment) OS=Homo sapiens GN=BCLAF1 PE=2 SV=1 - [Q6DCA8_HUMAN]	5.36	3.24	2	2	ETGYVVERPSTTK<br>SAAMTLNER
A5D904	RPS9 protein (Fragment) OS=Homo sapiens GN=RPS9 PE=2 SV=1 - [A5D904_HUMAN]	5.19	15.04	2	2	LFEGNALLR<br>LDYILGLK
O60701-2	Isoform 2 of UDP-glucose 6-dehydrogenase OS=Homo sapiens GN=UGDH - [UGDH_HUMAN]	3.92	4.68	2	2	LAANAFLAQR

					VTVVDVNESR

6 hpi

Accession	Description	Score	Coverage	Unique Peptides	Peptides	Identified sequence
G7NBW9	Glutathione S-transferase P (Fragment) OS=Macaca mulatta GN=EGK_05927 PE=3 SV=1 - [G7NBW9_MACMU]	68.51	45.71	5	8	EAALVDmVNDGVEDLR
						EAALVDMVNDGVEDLR
						FQDGDLTLYQSNTFLR
						mLLADQGQSWKEEVVTMETWQEGSLK
						YLSLIYTNYEAGKDDYVK
						MLLADQGQSWK
						YLSLIYTNYEAGK
						AScLYGQLPK
						TLGLYGKDQR
Q5R1W8	Vimentin OS=Pan troglodytes GN=VIM PE=2 SV=4 - [VIME_PANTR]	63.91	45.49	4	18	LQEEMLQREEAENTLQSFR
						EMEENFAVEAANYQDTIGR
						KVESLQEEIAFLKK
						LQDEIQDMKEEMAR
						LLQDSVDFSLADAINTEFK
						DGQVINETSQHHDDLE
						TNEKVELQELNDR
						QDVDNASLAR
						FADLSEAANR
						EEAENTLQSFR
						SLYASSPGGVYATR
						NLQEAEEWYK
						LGDLYEEEMR
						QVQSLTcEVDALK
						LQEEMLQR
						ETNLDSLPLVDTHSK
						LLEGEESR
						GTNESLER
F7HSL0	Carbonyl reductase [NADPH] 1 OS=Callithrix jacchus GN=CBR1 PE=2 SV=1 - [F7HSL0_CALJA]	56.71	35.02	2	8	GQAAVQQLQAEGLSPR
						SETITEEELVGLMNK
						FRSETITEEELVGLMNK
						ILLNAccPGWVR
						EYGGLDVLVNNAGIAFK
						SETITEEELVGLmNK
						VADPTPFHIQAEVTMK
						TDmAGPNATK
						ScSPELQQK
Q4R7H5	Elongation factor 1-gamma OS=Macaca fascicularis GN=EEF1G PE=2 SV=1 - [EF1G_MACFA]	23.66	17.62	7	7	KAAAPAPEEEMDEcEQALAAEPK
						ALIAAQYSGAQVR
						KLDPGSEETQTLVR
						STFVLDEFKR
						LDPGSEETQTLVR
						ILGLLDAHLK
						FAETQPK
U3BEK0	Tubulin beta-4B chain OS=Callithrix jacchus GN=TUBB4B PE=2 SV=1 - [U3BEK0_CALJA]	17.77	15.06	1	6	EVDEQMLNVQNK
						YLTVAAVFR
						INVYYNEAAGNK
						AILVDLEPGTMDSVR
						LAVNMVPFPR
						TAVcDIPPR
Q5T6W5	Heterogeneous nuclear ribonucleoprotein K OS=Homo sapiens GN=HNRNPK PE=1 SV=1 - [Q5T6W5_HUMAN]	14.61	15.89	5	5	GSYGDLGGPIITTQVTIPK
						GSDFDcELR
						LFQEccPHSTDR

					TDYNASVSVPDSSGPER<br>IDEPLEGSEDR
F6ZXW5	Glyceraldehyde-3-phosphate dehydrogenase OS=Macaca mulatta GN=GAPDH PE=3 SV=1 - [F6ZXW5_MACMU]	14.24	19.80	4	4	VPTANVSVVDLTcR<br>IVSNAScTTNcLAPLAK<br>VVDLMAHMASKE<br>GALQNIIPASTGAAK
Q2VPJ6	HSP90AA1 protein (Fragment) OS=Homo sapiens GN=HSP90AA1 PE=1 SV=1 - [Q2VPJ6_HUMAN]	12.05	8.03	1	4	GVVDSEDLPLNISR<br>DQVANSAFVER<br>YIDQEELNK<br>TLTIVDTGIGmTK
B7Z4V2	cDNA FLJ51907, highly similar to Stress-70 protein, mitochondrial OS=Homo sapiens PE=2 SV=1 - [B7Z4V2_HUM	10.33	7.37	4	4	QAASSLQQASLK<br>QAVTNPNNTFYATK<br>VLENAEGAR<br>TTPSVVAFTADGER
B4DNH8	Annexin OS=Homo sapiens PE=2 SV=1 - [B4DNH8_HUMAN]	10.09	16.49	3	3	TPAQYDASELK<br>TNQELQEINR<br>SLYYYIQQDTK
J3KTJ8	60S ribosomal protein L26 (Fragment) OS=Homo sapiens GN=RPL26 PE=4 SV=4 - [J3KTJ8_HUMAN]	9.01	26.04	4	4	KDDEVQVVR<br>DDEVQVVR<br>YVIYIER<br>FNPFVTSDR
B4DEF7	cDNA FLJ60062, highly similar to 78 kDa glucose-regulated protein OS=Homo sapiens PE=2 SV=1 - [B4DEF7_HU	8.57	14.39	2	3	NQLTSNPENTVFDAK<br>VEIIANDQGNR<br>ITPSYVAFTPEGER
A0A024R9E2	Poly(A) binding protein, cytoplasmic 1, isoform CRA_c OS=Homo sapiens GN=PABPC1 PE=4 SV=1 - [A0A024R9E	8.15	9.31	3	3	KEFSPFGTITSAK<br>SLGYAYVNFQQPADAER<br>SGVGNIFIK
F8VTQ5	Heterogeneous nuclear ribonucleoprotein A1 (Fragment) OS=Homo sapiens GN=HNRNPA1 PE=1 SV=1 - [F8VTQ	7.80	17.93	2	2	LFIGGLSFETTDESLR<br>IEVIEIMTDR
I7GK47	Pyruvate kinase OS=Macaca fascicularis PE=2 SV=1 - [I7GK47_MACFA]	7.63	6.15	3	3	VNFAMNVGK<br>GSGTAEVELKK<br>GSGTAEVELK
Q7KYM9	ORF protein OS=Homo sapiens GN=ORF PE=2 SV=1 - [Q7KYM9_HUMAN]	7.36	6.84	3	3	QGGGGGGGSVPGIER<br>FESPEVAER<br>MGPAMGPALGAGIER
D3DS95	HCG21173, isoform CRA_a OS=Homo sapiens GN=hCG_21173 PE=4 SV=1 - [D3DS95_HUMAN]	6.93	32.98	2	2	cTGGEVGATSALAPK<br>QAQIEVVPSASALIIK
I7GH78	Macaca fascicularis brain cDNA clone: QmoA-11140, similar to human DEAD (Asp-Glu-Ala-Asp) box polypeptide 1<br>transcript variant 1, mRNA, RefSeq: NM_006386.3 OS=Macaca fascicularis PE=2 SV=1 - [I7GH78_MACFA]	6.91	6.31	2	2	GDGPIcLVLAPTR<br>APILIATDVASR
Q15084-3	Isoform 3 of Protein disulfide-isomerase A6 OS=Homo sapiens GN=PDIA6 - [PDIA6_HUMAN]	6.77	7.32	2	2	LAAVDATVNQVLASR<br>GSTAPVGGGAFPTIVER
Q6DCA8	BCLAF1 protein (Fragment) OS=Homo sapiens GN=BCLAF1 PE=2 SV=1 - [Q6DCA8_HUMAN]	6.61	4.56	2	2	SQEEPKDTFEHDPSESIDEFNK<br>SAAMTLNER<br>SAAmTLNER
B7Z5E7	cDNA FLJ51046, highly similar to 60 kDa heat shock protein, mitochondrial OS=Homo sapiens PE=2 SV=1 - [B7Z5	6.16	4.64	2	2	TVIIEQSWGSPK<br>VGGTSDVEVNEK
E9PKZ0	60S ribosomal protein L8 (Fragment) OS=Homo sapiens GN=RPL8 PE=1 SV=1 - [E9PKZ0_HUMAN]	6.08	16.59	3	3	ASGNYATVISHNPETK<br>AVVGVVAGGGR<br>AVDFAER
A2A3R7	40S ribosomal protein S6 OS=Homo sapiens GN=RPS6 PE=1 SV=1 - [A2A3R7_HUMAN]	6.06	29.67	2	2	MATEVAADALGEEWK<br>LNISFPATGcQK
O60701-2	Isoform 2 of UDP-glucose 6-dehydrogenase OS=Homo sapiens GN=UGDH - [UGDH_HUMAN]	5.86	4.68	2	2	VTVVDVNESR<br>LAANAFLAQR
P62263	40S ribosomal protein S14 OS=Homo sapiens GN=RPS14 PE=1 SV=3 - [RS14_HUMAN]	5.60	15.89	2	2	TPGPGAQSALR<br>IEDVTPIPSDSTR
A5D904	RPS9 protein (Fragment) OS=Homo sapiens GN=RPS9 PE=2 SV=1 - [A5D904_HUMAN]	5.45	14.16	2	2	IEDFLER

						LFEGNALLR
A0A0A0MSI0	Peroxiredoxin-1 (Fragment) OS=Homo sapiens GN=PRDX1 PE=4 SV=1 - [A0A0A0MSI0_HUMAN]	5.31	10.53	2	2	TIAQDYGVLK<br>ADEGISFR
E9PPU1	40S ribosomal protein S3 OS=Homo sapiens GN=RPS3 PE=1 SV=1 - [E9PPU1_HUMAN]	4.86	13.92	2	2	DEILPTTPISEQK<br>TEIIILATR
G7PQL6	L-lactate dehydrogenase OS=Macaca fascicularis GN=EGM_05805 PE=3 SV=1 - [G7PQL6_MACFA]	4.63	5.42	2	2	SADTLWGIQK<br>FIVPNVVK
H0YFC6	GTP-binding nuclear protein Ran (Fragment) OS=Homo sapiens GN=RAN PE=1 SV=1 - [H0YFC6_HUMAN]	3.94	18.45	2	2	HLTGEFEK<br>LVLVGDGGTGK

18 hpi

Accession	Description	Score	Coverage	Unique Peptides	Peptides	Identified sequence
F7HSL0	Carbonyl reductase [NADPH] 1 OS=Callithrix jacchus GN=CBR1 PE=2 SV=1 - [F7HSL0_CALJA]	125.97	31.77	1	7	GQAAVQQLQAEGLSPR<br>SETITEEELVGLMNK<br>EYGGLDVLVNNAGIAFK<br>ILLNAccPGWVR<br>VADPTPFHIQAEVTMK<br>FRSETITEEELVGLMNK<br>SETITEEELVGLmNK<br>VADPTPFHIQAEVTmK<br>TDmAGPNATK
Q28514	Glutathione S-transferase P OS=Macaca mulatta GN=GSTP1 PE=2 SV=3 - [GSTP1_MACMU]	121.77	49.52	5	8	EAALVDmVNDGVEDLR<br>FQDGDLTLYQSNTFLR<br>EAALVDMVNDGVEDLR<br>YLSLIYTNYEAGKDDYVK<br>AFLASPEHVNLPINGNGK<br>MLLADQGQSWK<br>PFETLLSQNQGGK<br>mLLADQGQSWK<br>MPPYTVVYFPVR<br>YLSLIYTNYEAGK
B3KML9	cDNA FLJ11352 fis, clone HEMBA1000020, highly similar to Tubulin beta-2C chain OS=Homo sapiens PE=2 SV=1 - [B3KML9_HUMAN]	38.02	16.37	1	6	IMNTFSVVPSPK<br>EVDEQmLNVQNK<br>EVDEQMLNVQNK<br>INVYYNEATGGK<br>LAVNMVPFPR<br>ISEQFTAmFR<br>TAVcDIPPR
Q4R7H5	Elongation factor 1-gamma OS=Macaca fascicularis GN=EEF1G PE=2 SV=1 - [EF1G_MACFA]	32.29	13.50	6	6	WFLTcINQPQFR<br>ALIAAQYSGAQVR<br>KLDPGSEETQTLVR<br>ILGLLDAHLK<br>STFVLDEFKR<br>LDPGSEETQTLVR
B7Z5E7	cDNA FLJ51046, highly similar to 60 kDa heat shock protein, mitochondrial OS=Homo sapiens PE=2 SV=1 - [B7Z5	19.78	8.70	4	4	VGGTSDVEVNEK<br>VGLQVVAVK<br>TVIIEQSWGSPK<br>GYISPYFINTSK
B4DMA2	cDNA FLJ54023, highly similar to Heat shock protein HSP 90-beta OS=Homo sapiens PE=2 SV=1 - [B4DMA2_HU	18.83	9.91	5	5	NPDDITQEEYGEFYK<br>SLTNDWEDHLAVK<br>YHTSQSGDEMTSLSEYVSR<br>YIDQEELNK<br>ADLINNLGTIAK
A6NMY6	Putative annexin A2-like protein OS=Homo sapiens GN=ANXA2P2 PE=5 SV=2 - [AXA2L_HUMAN]	16.24	11.21	3	3	SLYYYIQQDTK<br>TDLEKDIISDTSGDFRK<br>TNQELQEINR

P68363-2	Isoform 2 of Tubulin alpha-1B chain OS=Homo sapiens GN=TUBA1B - [TBA1B_HUMAN]	12.45	14.03	3	3	TIGGGDDSFNTFFSETGAGK<br>LISQIVSSITASLR<br>SIQFVDWcPTGFK
Q9BS10	Similar to ribosomal protein S8 (Fragment) OS=Homo sapiens PE=2 SV=1 - [Q9BS10_HUMAN]	11.66	27.50	2	2	ISSLLEEQFQQGK<br>ADGYVLEGK
Q6DCA8	BCLAF1 protein (Fragment) OS=Homo sapiens GN=BCLAF1 PE=2 SV=1 - [Q6DCA8_HUMAN]	10.23	4.56	3	3	ETGYVVERPSTTK<br>SAAMTLNER<br>TIAPQNAPR
F7GV16	Elongation factor 1-alpha OS=Macaca mulatta GN=LOC722466 PE=3 SV=1 - [F7GV16_MACMU]	8.33	8.86	3	3	IGGIGTVPVGR<br>YYVTIIDAPGHR<br>LPLQDVYK
E9PG15	14-3-3 protein theta (Fragment) OS=Homo sapiens GN=YWHAQ PE=1 SV=1 - [E9PG15_HUMAN]	7.93	17.45	2	2	AVTEQGAELSNEER<br>YLAEVAcGDDRK
G7P1R3	Testis-specific adriamycin sensitivity protein (Fragment) OS=Macaca fascicularis GN=EGM_12506 PE=4 SV=1 [G7P1R3_MACFA]	7.83	8.14	2	2	AVVcQESDLPDELLYGR<br>SGNYPSSLSNETDR
H9FCA8	Histone H4 (Fragment) OS=Macaca mulatta GN=HIST1H4K PE=2 SV=1 - [H9FCA8_MACMU]	7.81	37.04	3	3	DNIQGITKPAIR<br>VFLENVIR<br>DAVTYTEHAK
A0A0A0MSI0	Peroxiredoxin-1 (Fragment) OS=Homo sapiens GN=PRDX1 PE=4 SV=1 - [A0A0A0MSI0_HUMAN]	7.33	14.62	3	3	TIAQDYGVLK<br>ADEGISFR<br>SVDETLR
B5MCT8	40S ribosomal protein S9 OS=Homo sapiens GN=RPS9 PE=3 SV=1 - [B5MCT8_HUMAN]	7.02	12.23	2	2	LFEGNALLR<br>LIGEYGLR
P62263	40S ribosomal protein S14 OS=Homo sapiens GN=RPS14 PE=1 SV=3 - [RS14_HUMAN]	6.36	15.89	2	2	IEDVTPIPSDSTR<br>TPGPGAQSALR
O60701-2	Isoform 2 of UDP-glucose 6-dehydrogenase OS=Homo sapiens GN=UGDH - [UGDH_HUMAN]	6.25	5.39	2	2	VTVVDVNESR<br>VLIGGDETPEGQR
Q5T6W2	Heterogeneous nuclear ribonucleoprotein K (Fragment) OS=Homo sapiens GN=HNRNPK PE=1 SV=1 - [Q5T6W2_	6.01	7.39	2	2	NTDEMVELR<br>GSYGDLGGPIITTQVTIPK
K7EMA7	60S ribosomal protein L23a OS=Homo sapiens GN=RPL23A PE=3 SV=1 - [K7EMA7_HUMAN]	5.59	34.29	2	2	VNTLIRPDGEK<br>LAPDYDALDVANK
F8W7C6	60S ribosomal protein L10 OS=Homo sapiens GN=RPL10 PE=1 SV=2 - [F8W7C6_HUMAN]	5.59	13.50	2	2	FNADEFEDMVAEK<br>MLScAGADR
V5XJZ0	Ribosomal protein S4, X-linked (Fragment) OS=Macaca fascicularis GN=RPS4X PE=4 SV=1 - [V5XJZ0_MACFA]	5.55	20.00	2	2	VNDTIQIDLETGK<br>LSNIFVIGK
C9JXB8	60S ribosomal protein L24 OS=Homo sapiens GN=RPL24 PE=1 SV=1 - [C9JXB8_HUMAN]	5.38	17.36	2	2	AITGASLADIMAK<br>cESAFLSK
M0R0P7	60S ribosomal protein L18a OS=Homo sapiens GN=RPL18A PE=1 SV=1 - [M0R0P7_HUMAN]	4.80	19.71	2	2	SSGEIVYcGQVFEK<br>DLTTAGAVTQcYR
W5X314	Lamin A/C (Fragment) OS=Homo sapiens PE=4 SV=1 - [W5X314_HUMAN]	4.78	26.67	2	2	SGAQASSTPLSPTR<br>ITESEEVVSR

Supplementary table 2. To each sample, table shows the pathway where the proteins were enriched (enriched pathway), the proteins of the Mass Spectometry analysis founded in that pathway (members) and the q-value.

Mock

Enriched pathway	Members	q-value
Ribosome	RPL37A; RPL8; RPS9; RPS14	1.39E-05
Peptide chain elongation	RPL37A; RPL8; RPS9; RPS14	9.07E-06
Translation	RPL37A; RPL8; RPS9; RPS14	2.23E-05
rRNA processing	RPS9; RPS14	0.0021015
mRNA Processing	HNRNPK; YBX1	0.0063127
NRF2 pathway	CBR1; GSTM5	0.0073812

6 hpi

Enriched pathway	Members	q-value
Translation	RPL8; RPS14; PABPC1; RPL26; RPS6; RPS3; EEF1G; RPS9	4.41E-09
Eukaryotic Translation Elongation	RPL8; RPS14; EEF1G; RPL26; RPS6; RPS3; RPS9	4.41E-09
Ribosomal Proteins	RPL8; RPS14; RPL26; RPS6; RPS3; RPS9	2.35E-08
Translation initiation complex formation	RPS6; RPS14; RPS3; RPS9; PABPC1	2.15E-07
rRNA processing	RPS6; RPS9; RPS14	0.0003499
NRF2 pathway	HSP90AA1; CBR1; PRDX1	0.0030956
Centrosome maturation	TUBB4B; HSP90AA1	0.0128055
G2/M Transition	TUBB4B; HSP90AA1	0.0146115

18 hpi

Enriched pathway	Members	q-value
Translation	RPL23A; RPS14; EEF1G; RPL18A; RPL10; RPL24; RPS4X; RPS9	1.82E-10
Peptide chain elongation	RPL23A; RPS14; RPL24; RPL18A; RPL10; RPS4X; RPS9	1.82E-10
Ribosomal Proteins	RPL23A; RPS14; RPL24; RPL18A; RPL10; RPS4X; RPS9	1.54E-10
Translation initiation complex formation	RPS4X; RPS9; RPS14	0.0001515
Viral carcinogenesis	HIST1H4K; HNRNPK; YWHAQ	0.0033259
rRNA processing	RPS9; RPS14	0.0044083
G2/M Checkpoints	HIST1H4K; YWHAQ	0.0125353
